# Design, Synthesis, Anticancer Screening, and Mechanistic Study of Spiro-N-(4-sulfamoyl-phenyl)-1,3,4-thiadiazole-2-carboxamide Derivatives

**DOI:** 10.3390/ijms26020863

**Published:** 2025-01-20

**Authors:** Ahmed M. El-Saghier, Hamada Hashem, Sherif A. Maher, Souhaila S. Enaili, Abdullah Alkhammash, Stefan Bräse, Hossameldin A. Aziz

**Affiliations:** 1 Department of Chemistry, Faculty of Science, Sohag University, Sohag 82524, Egypt; 2 Department of Pharmaceutical Chemistry, Faculty of Pharmacy, Sohag University, Sohag 82524, Egypt; 3 Department of Biochemistry, Faculty of Pharmacy, New Valley University, New Valley 72511, Egypt; sherif.ali87@pha.nvu.edu.eg; 4 Department of Chemistry, Faculty of Science, University of Zawia, Az Zawiyah 16418, Libya; s.enaili@zu.edu.ly; 5 Department of Pharmacology, College of Pharmacy, Shaqra University, Shaqra 11961, Saudi Arabia; alkhammash@su.edu.sa; 6 Institute for Biological and Chemical System, Karlsruhe Institute of Technology, 76131 Karlsruhe, Germany; 7 Department of Pharmaceutical Chemistry, Faculty of Pharmacy, New Valley University, New Valley 72511, Egypt; hossamaziz85@pha.nvu.edu.eg

**Keywords:** anticancer, thiadiazole, carbonic anhydrases inhibitor, molecular docking

## Abstract

The present study aims to create spiro-N-(4-sulfamoyl-phenyl)-1,3,4-thiadiazole-2-carboxamide derivatives with anticancer activities. The in vitro anticancer evaluation showed that only the novel spiro-acenaphthylene tethered-[1,3,4]-thiadiazole (compound **1**) exhibited significant anticancer efficacy as a selective inhibitor of tumor-associated isoforms of carbonic anhydrase. Compound **1** demonstrated considerable efficacy against the renal RXF393, colon HT29, and melanoma LOX IMVI cancer cell lines, with IC_50_ values of 7.01 ± 0.39, 24.3 ± 1.29, and 9.55 ± 0.51 µM, respectively. In comparison, doxorubicin exhibited IC_50_ values of 13.54 ± 0.82, 13.50 ± 0.71, and 6.08 ± 0.32 µM for the corresponding cell lines. Importantly, compound **1** exhibited lower toxicity to the normal WI 38 cell line than doxorubicin, with IC_50_ values of 46.20 ± 2.59 and 18.13 ± 0.93 µM, respectively, indicating greater selectivity of the target compound compared to the standard anticancer agent doxorubicin. Also, mechanistic experiments demonstrated that compound **1** exhibits inhibitory activity against human carbonic anhydrase hCA IX and XII, with IC_50_ values of 0.477 ± 0.03 and 1.933 ± 0.11 μM, respectively, indicating enhanced selectivity for cancer-associated isoforms over cytosolic isoforms hCA I and II, with IC_50_ values of 7.353 ± 0.36 and 12.560 ± 0.74 μM, respectively. Cell cycle studies revealed that compound **1** caused G1 phase arrest in RXF393 cells, and apoptosis experiments verified a substantial induction of apoptosis with significant levels of early and late apoptosis, as well as necrosis (11.69%, 19.78%, and 3.66%, respectively), comparable to those induced by the conventional cytotoxic agent doxorubicin, at 9.91%, 23.37%, and 6.16%, respectively. Molecular docking experiments confirmed the strong binding affinity of compound **1** to the active sites of hCA IX and XII, highlighting significant interactions with zinc-binding groups and hydrophobic residues. These findings underscore the target compound’s potential as a viable anticancer agent via targeting CA.

## 1. Introduction

Cancer presents a substantial global health problem, with millions of new cases diagnosed each year and it being one of the leading causes of death worldwide [1]. The condition can appear in various ways, impacting almost any organ or tissue, and presents a substantial worldwide health problem [2]. A combination of genetic predisposition, environmental factors, and lifestyle choices frequently influences cancer development [3]. The reduction in oxygen levels in the microenvironment of solid cancer results in a condition called hypoxia [4,5]. To survive and flourish in these circumstances, cancer cells utilize glycolysis and enhance the production of particular enzymes to decrease the external pH [6,7,8].

hCA IX isoform, among these enzymes, is increased by tumor cells in response to hypoxia and is a well-established enzyme in renal cell carcinoma [9,10]. This helps the cells adapt to the acidic conditions caused by low oxygen levels, ultimately promoting the growth of cancer cells [11,12,13,14]. In contrast, different solid tumors such as breast, lung, and cervical cancers highly stimulate hCA XII [15,16]. These enzymes use zinc as a crucial co-factor to regulate the external pH by facilitating the reversible conversion between bicarbonate ions and carbon dioxide [15,16]. At the molecular level, all isoforms of human carbonic anhydrase have a structurally conserved active site. This active site is characterized by a cone-shaped pocket that contains a zinc ion coordinated with three specific amino acid residues (His 94, His 96, and His 119) and water [17,18]. This active site’s outer edge comprises hydrophilic or hydrophobic regions [19]. These regions differ in their degree of hydrophobicity and polarity among different hCA isoforms [20,21]. So, hCA inhibitors (CAIs) have a zinc-binding group (ZBG) that is needed to connect with the zinc ion in the active site [22,23]. Sulfonamide-containing compounds work very well as hCAIs, but they do not pick and choose which hCA isoforms to target, which can cause unwanted side effects like paresthesia, fatigue, and decreased libido [24,25,26]. Because the active sites of different CA isoforms are very similar, it has been hard to make an inhibitor that only targets certain diseases [27,28].

**Figure 1:**
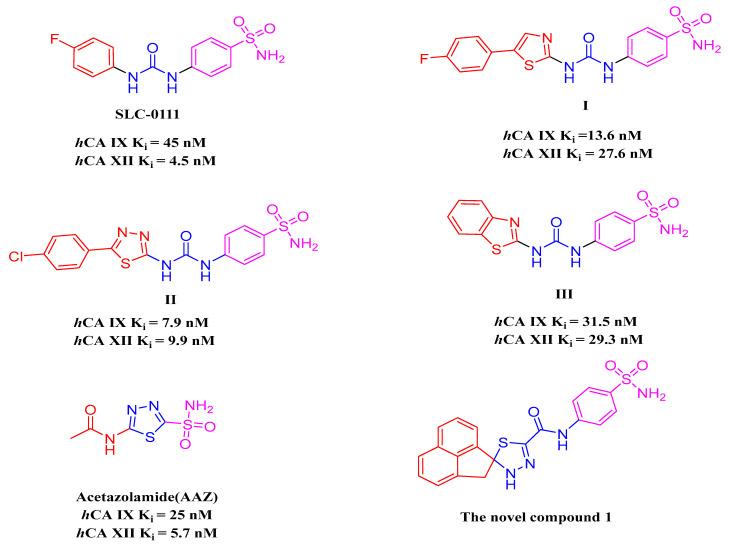
Chemical structures of SLC-0111, its reported analogs, and the novel compound **1**.

To tackle this selectivity problem, the “tail approach” has emerged as a promising strategy [29,30]. This method entails adding different substituted phenyl or heterocyclic structures to the aromatic sulfonamide ring [31]. These structures interact with specific hydrophilic/hydrophobic residues in the outer regions of the isoform’s active site [32]. The tail approach was used to make selective hCAIs, and this led to the discovery of SLC-0111, which is the first selective CAI for the hCA IX isoform and is currently in clinical trial phases I and II. This compound shows promise in treating patients with advanced solid tumors, as shown in Figure 1 [33,34]. Multiple analogs of SLC-0111 have been created by substituting its 4-fluorophenyl tail with various chemical frameworks [35]. Compound **I** was synthesized by substituting the 4-fluorophenyl group of SLC-0111 with 5-(4-fluorophenyl) thiazole [36]. Compounds **II** and **III** were synthesized by substituting the tail of SLC-0111 with benzothiazole [36] and a substituted 1,3,5-triazine moiety [25], respectively, as shown in Figure 1.

**Figure 2:**
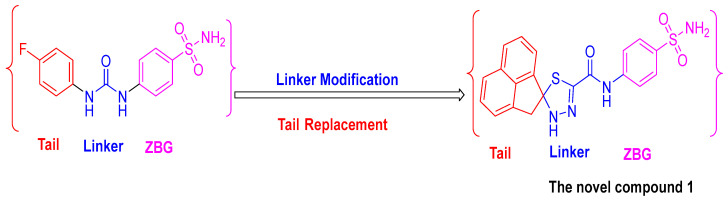
Designing a novel carbonic anhydrase inhibitor.

Inspired by these findings, this inquiry uncovers the development of a novel chemical designed to replicate the structure of SLC-0111. Our research aims to identify distinctive and precise inhibitors of tumor-associated carbonic anhydrases, as seen in Figure 2. Firstly, we substituted the para-fluorophenyl tail in SLC-0111 with a spiro acenaphthylene moiety in compound **1**, which led to the creation of a newly synthesized acenaphthylene-linked [1,3,4] thiadiazol-based counterpart of SLC-0111.

## 2. Result and Discussion

### 2.1. Chemistry

**Scheme 1:**
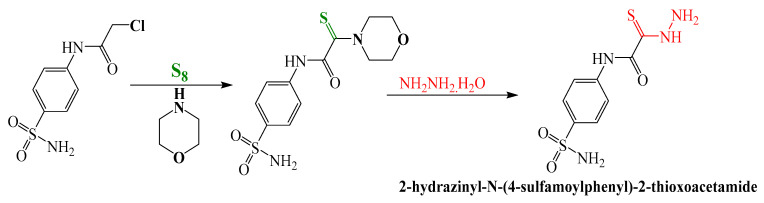
Synthesis of the intermediate compound: 2-hydrazinyl-N-(4-sulfamoylphenyl)-2-thioxoacetamide.

The key intermediate compound 2-hydrazinyl-N-(4-sulfamoylphenyl)-2-thioxoacetamide was synthesized by the reaction of 2-chloro-N-sulfamoylphenyl acetamide with morpholine and sulfur, followed by a reaction with hydrazine hydrate as reported in Scheme 1 [37]. This intermediate compound was confirmed by IR and NMR spectroscopic techniques (Figures S1 and S2).

**Scheme 2:**
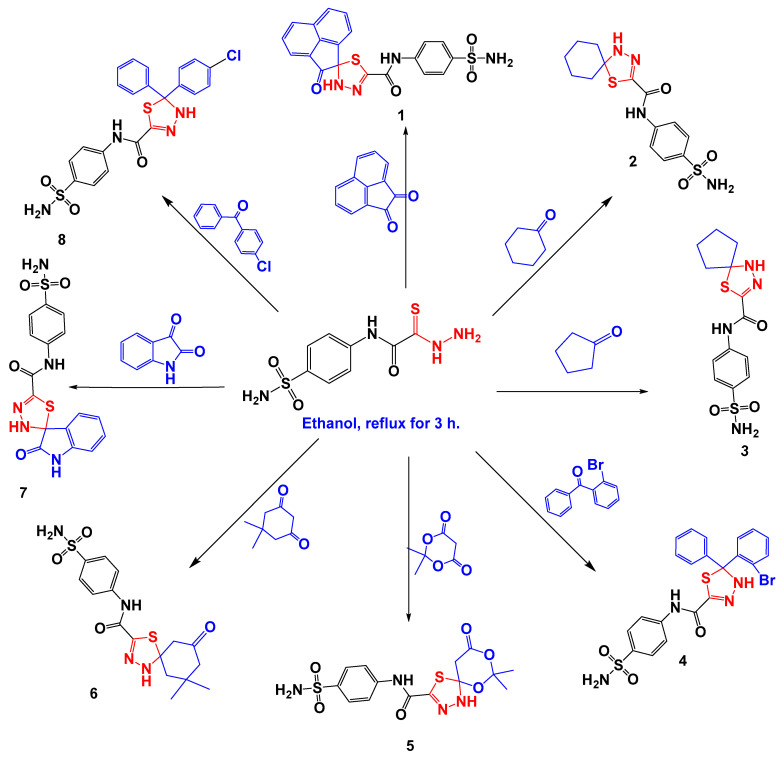
Synthesis of the target compounds **1**–**8** under green condition in ethanol at room temperature.

Compound **1**, a novel spiro-heterocycle, was synthesized by a reaction of equimolar amounts of 2-hydrazinyl-N-(4-sulfamoylphenyl)-2-thioxoacetamide (compound **1**) and acenaphthylene-1,2-dione under a green condition in ethanol at room temperature with a good yield (87%) as illustrated in Scheme 2. Other reported compounds **2**–**8** were synthesized as reported and confirmed by their melting points [37]. A ^1^H NMR spectrum of the novel compound **1** showed three singlets corresponding to 2(NH) in addition to NH_2_. Also, the ^1^H NMR spectrum showed ten aromatic protons at the expected chemical shifts (Figure S3). ^13^CNMR showed the appearance of two carbonyls at 159.0 and 182.0 ppm corresponding to the amidic and the ketonic carbon. Also, spiro carbon at 86.2 ppm and other aromatic carbons appear at the expected chemical shift (Figure S4). Also, the mass spectrometry confirms the structure (Figure S5).

### 2.2. Biology

#### 2.2.1. One-Dose Anticancer Screening of the Target Compound **1** (NCI, Bethesda, MD, USA)

**Figure 3:**
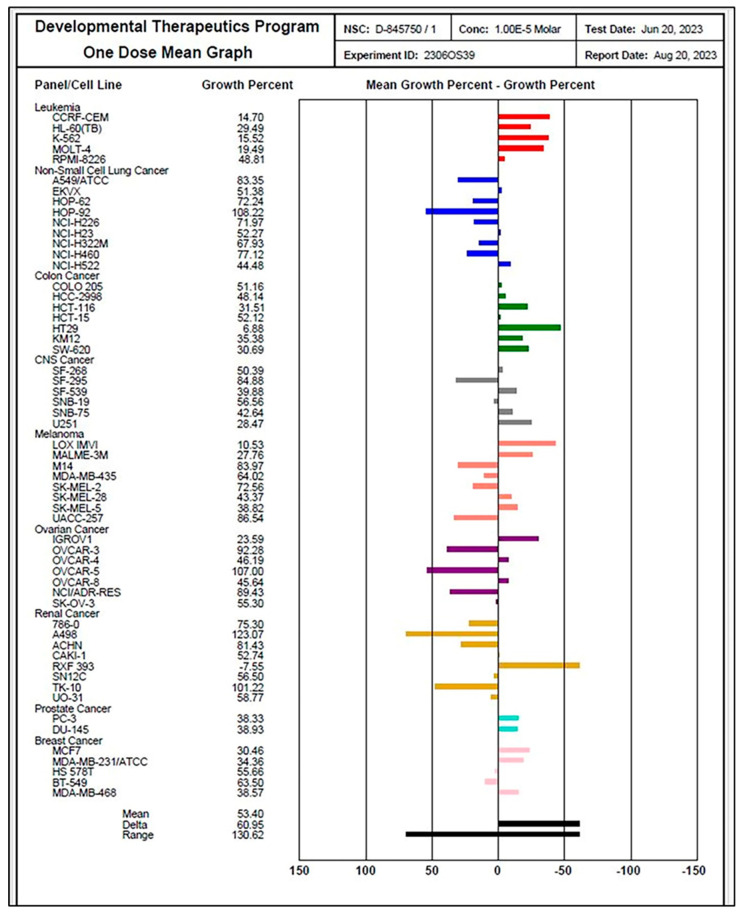
One-dose anticancer screening of compound **1** (NCI, USA).

Following the anticancer screening guidelines controlled by the National Cancer Institute (NCI), USA drug evaluation section [38], all the synthesized compounds were examined against 60 cancer cell lines at a single concentration of 10 µM. The results of the NCI anticancer screening indicated that only compound **1** exhibited significant anticancer activity among the eight compounds tested as shown in Figure 3 (results are shown in detail in the Supplementary Data Figures S6–S13).

**Figure 4:**
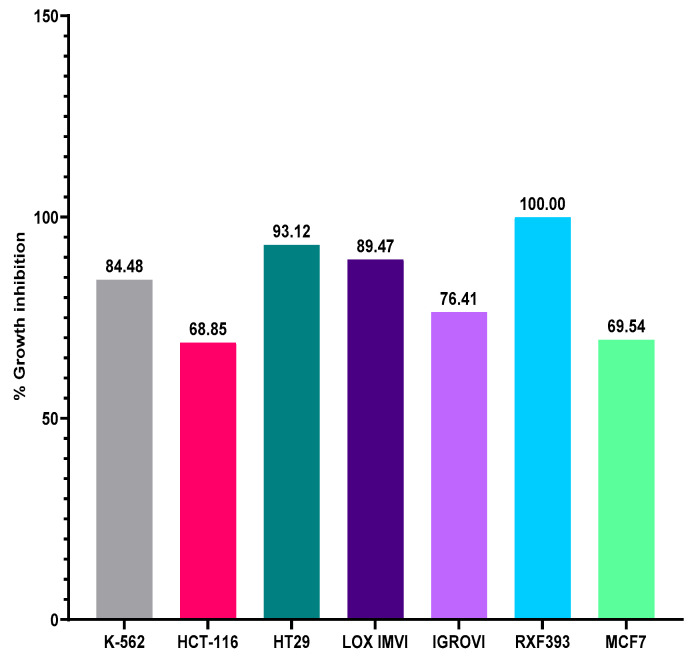
The important results of one-dose anticancer screening of the compound **1** against K-562, HCT-116, HT29, LOX IMVI, IGROV1, RXF393, and MCF7 (NCI, USA) in single-dose assay (10 µM).

The results in Figure 3 and Figure 4 demonstrate the potent anticancer activity of compound **1** against the melanoma LOX IMVI, colon HT29, and renal RXF393 cell lines with growth inhibition percentages of 89.47, 93.12, and 100, respectively. Good activity was observed against leukemic cancer cell lines MOLT-4, CCRF-CEM, and K-562 with growth inhibition percentages of 80.51, 85.30, and 84.48, respectively. Also, the target compound showed moderate anticancer activities against the leukemic HL-60, colon HCT-116, CNS U251, melanoma MALME-3M, ovarian IGROVE 1, and breast MCF-7 cancer cell lines with growth inhibition percent over 68%. The notable anticancer efficacy of compound **1**, particularly against the renal cancer cell line RXF393, prompts us to conduct a more in-depth mechanistic investigation of this molecule, encompassing cell cycle analysis, apoptosis assessment, and carbonic anhydrase inhibition assays.

#### 2.2.2. Cell Viability Assay of the Target Compound Against Melanoma LOX IMVI, Colon HT29, and Renal RXF393 Cancer Cell Lines in Addition to Normal Cell Line WI 38

**Figure 5:**
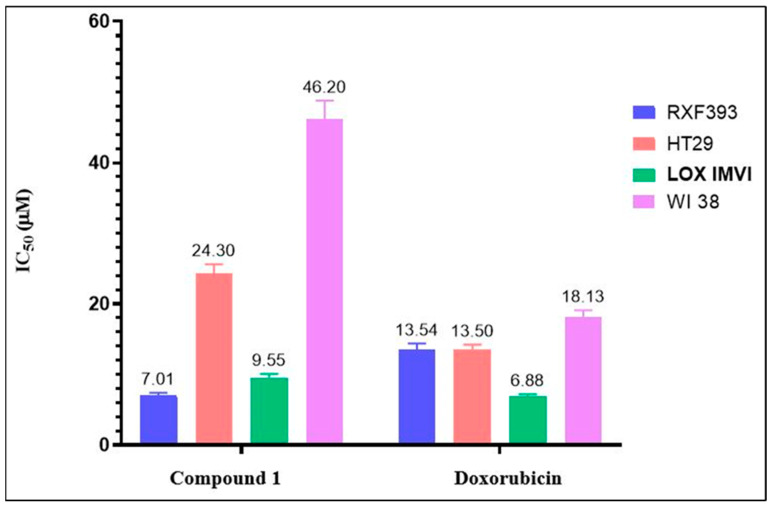
In vitro cytotoxicity expressed as half inhibitory concentration (IC_50,_ µM ± SD) of the target compound against melanoma LOX IMVI, renal RXF393, and colon HT29 cancer cell lines in comparison to the conventional doxorubicin.

Based on the results above, the target compound **1** was selected for IC_50_ determination against the most sensitive cell lines—the melanoma LOX IMVI colon HT29, and renal RXF393 cell lines in addition to normal cell line WI 38 compared to doxorubicin as a reference compound using the MTT assay [2] (Figure 5, Tables S1–S4). Screening results showed potent anticancer activity of compound **1** against RXF393 with an IC_50_ value of 7.01 ± 0.39 µM, which is nearly double the potency of doxorubicin with an IC_50_ value of 13.54 ± 0.82 µM. Also, the compound **1** has comparable activity to doxorubicin against LOX IMVI with IC_50_ values of 9.55 ± 0.51 and 6.08 ± 0.32 µM, respectively. Moreover, compound **1** showed moderate anticancer activity against the colon HT29 cell line with an IC_50_ value of 24.3 ± 1.29 µM, with lower potency than the reference compound doxorubicin, which has an IC_50_ value of 13.50 ± 0.71 µM (Figure 4). Also, compound **1** and the cytotoxic doxorubicin were investigated against the normal cell line WI 38 to demonstrate their selective action against the cancer cell line (Figure 5, Table S3). The results indicated that the target compound possesses a safety margin greater than the cytotoxic drug doxorubicin, with IC_50_ values of 46.20 ± 2.59 and 18.13 ± 0.93 µM, respectively. These results mean that compound **1** is more selective than the cytotoxic doxorubicin toward the cancer cell line with low harm toward the normal human cell line, which is crucial for developing new anticancer agents.

#### 2.2.3. Evaluation of Carbonic Anhydrase I, II, IV, and VII Inhibition

Human Carbonic anhydrase (hCA) inhibition assays were conducted on the newly synthesized target compound and the standard CA inhibitor, acetazolamide, using an in vitro CA inhibitory assay. The aim was to evaluate their effectiveness against cancer-associated isoforms, hCA IX and hCA XII, as well as the cytosolic isoforms hCA I and hCA II, in order to better understand the potential anticancer mechanism of compound **1**. The carbonic anhydrase assay results highlight significant differences in the IC_50_ values and selectivity ratios of compound **1** and acetazolamide for the tumor-associated isoforms hCA IX and hCA XII. Compound **1** showed moderate potency against hCA IX (IC_50_ = 0.477 μM) and hCA XII (IC_50_ = 1.933 μM), with markedly high selectivity ratios for hCA IX, including hCA I/hCA IX = 15.41 and hCA II/hCA IX = 26.33, indicating a strong preference for inhibiting hCA IX over other isoforms (Table 1). This selectivity suggests potential utility in targeting tumor-associated CAs while sparing non-tumor isoforms. In contrast, acetazolamide demonstrates much lower IC_50_ values for both tumor-associated isoforms (hCA IX = 0.105 μM and hCA XII = 0.029 μM), reflecting higher potency but significantly lower selectivity ratios, with hCA I/hCA IX = 3.49 and hCA II/hCA IX = 1.56 (Table 2 and Tables S4–S8). While acetazolamide is broadly effective, its lack of selectivity may result in off-target effects. Compound **1**’s higher selectivity for hCA IX and hCA XII suggests a more focused therapeutic profile, particularly for conditions such as cancer, where these isoforms are upregulated.

#### 2.2.4. Cell Cycle Analysis

**Figure 6:**
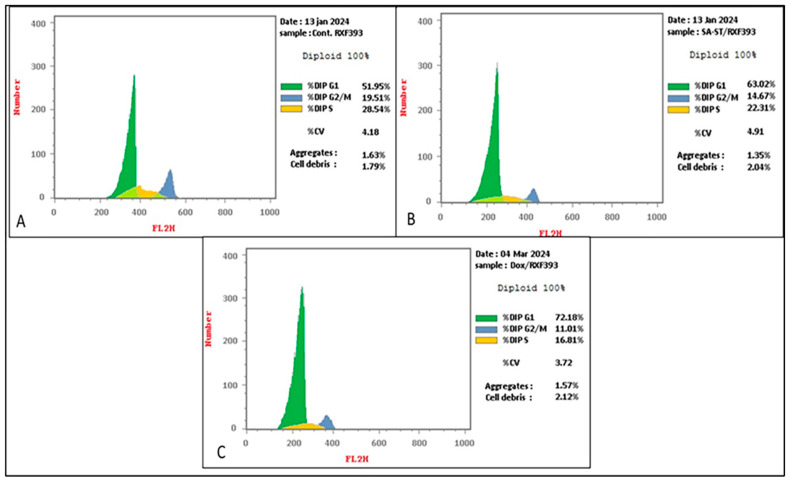
Effect of DMSO (control, (**A**)), target compound (IC_50_, 7.01 μM, (**B**)), and doxorubicin (IC_50_, 13.54 μM, (**C**)) on the accumulation of cells at different phases of the cell cycle of renal RXF-393 cancer cells.

**Figure 7:**
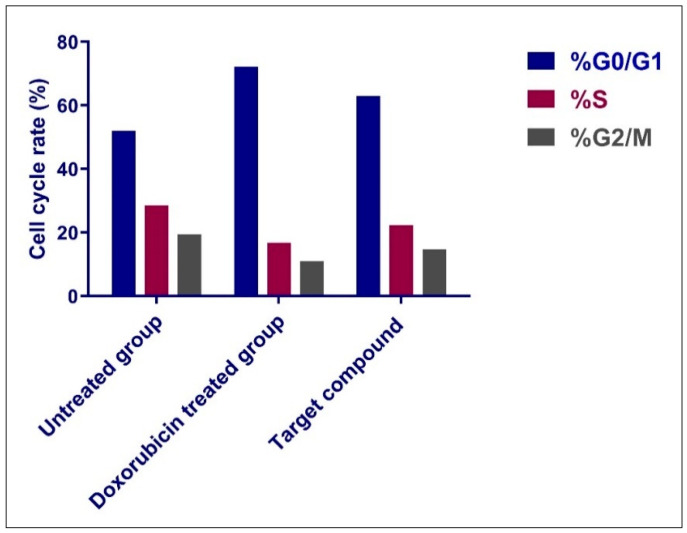
Analysis of cell cycle progression in RXF393 cells following treatment with 7.01 ± 0.39 µM of the target compound revealed cell cycle arrest at the G1/S phase. A two-way ANOVA test is employed to analyze significant differences.

Cell cycle regulatory systems are primarily responsible for regulating cell proliferation. In tumor cells, cell cycle arrest can decrease cell proliferation [39]. The impact of the target compound on cell cycle progression in the RXF393 cancer cell line was evaluated against doxorubicin as a positive control and untreated RXF393 as a negative control, utilizing a flow cytometry assay. Results showed that the percentage of cells in the G0-G1 phase increased from 51.95 to 63.02 when the renal RXF393 cancer cell line was treated with the target compound **1** at its previously measured IC_50_, suggesting the tendency of the target compound **1** to induce cell cycle arrest at the G1 phase (Table 2 and Figure 6 and Figure 7).

#### 2.2.5. Apoptosis Assay

**Figure 8:**
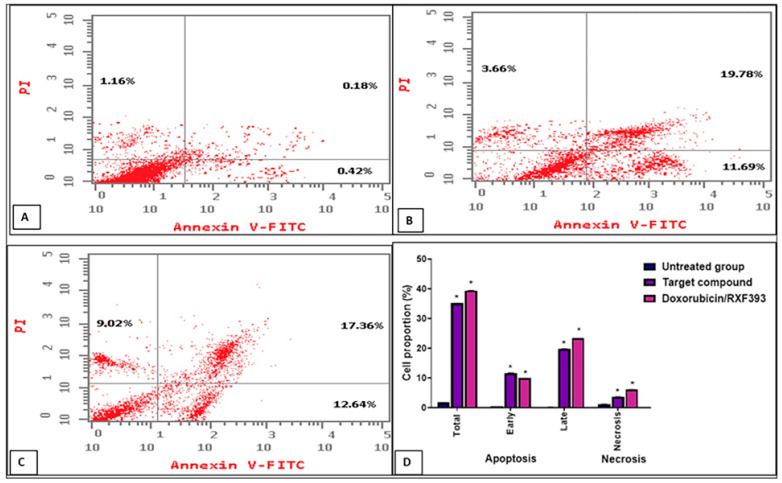
Flow cytometric dot plot for renal RXF393 cells treated with target compound, doxorubicin, and untreated cells after Annexin V-FITC/PI staining. Panel (**A**) untreated cells; panel (**B**) target compound **1**/RXF393 (IC_50_, 7.01 µM); panel (**C**) doxorubicin/RXF393 (IC_50_, 13.54 µM); and panel (**D**) bar graph of % cell proportion of early, late apoptotic, and necrotic cells. The four quadrants are designated as follows: Low Left (LL) for viable cells, Low Right (LR) for early apoptotic cells, Upper Left (UL) for necrotic cells, and Upper Right (UR) for late apoptotic cells. Bars indicate the means ± standard deviation (SD). Significant differences are assessed using one-way ANOVA (analysis of variance), followed by the Bonferroni post hoc test for multiple comparisons, and a two-way ANOVA test, where * *p* < 0.05 compared to the untreated group.

Programmed cell death, sometimes called “cellular suicide” or apoptosis, removes superfluous cells from healthy cells. Too little apoptosis, which can lead to malignant cells, is a disease-defining feature of cancer [40]. Apoptosis is a complicated process that involves numerous Routes. Apoptotic pathway abnormalities may not only encourage malignancy metamorphosis but it can also make tumor chemotherapy less effective [40]. The apoptotic capacity of the target compound **1** was examined to see whether its anticancer efficacy against the renal RXF393 cell line correlates with an enhancement of apoptosis and necrosis. The annexin assay analysis indicated that the treatment of the renal RXF393 cell line with the IC_50_ concentration of the target compound **1** resulted in significant levels of early and late apoptosis, as well as necrosis (11.69%, 19.78%, and 3.66%, respectively) comparable to those induced by the conventional cytotoxic agent doxorubicin, at 12.64%, 17.36%, and 9.02%, respectively (Table 3 and Figure 8). The apoptosis assay results explain that the target compound’s potent anticancer activity against the RXF393 cancer cell line is related to the induction of apoptosis in addition to CA inhibition.

#### 2.2.6. Analysis of Relative Gene Expression by Quantitative Real-Time PCR

**Figure 9:**
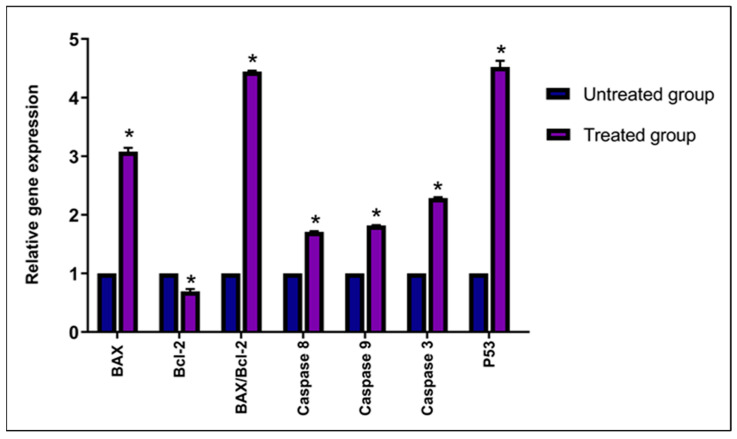
Relative expression levels of BAX, Bcl-2, the BAX/Bcl-2 ratio, and caspases (8, 9, 3), along with P53. Relative expression normalized to the internal control GAPDH. Bars denote the means ± standard deviation (SD). Significant differences are assessed using one-way ANOVA, followed by the Bonferroni post hoc test for multiple comparisons, and a two-way ANOVA, where * *p* < 0.05 indicates significance relative to the untreated group.

More importantly, apoptosis is assessed by evaluating the expression of apoptotic markers by qPCR. The apoptotic markers, BAX, caspases (3, 8, and 9), P53, and Bcl-2 relative gene expression were influenced through this study after treatment with the target compound **1**/RXF393 (Figure 8). The relative expression of BAX; caspases 3, 8, and 9; and P53 showed significant upregulation (*p* < 0.05) compared to the untreated cells. Meanwhile, Bcl-2 mRNA expression was significantly downregulated after treatment with compound 1/RXF393. Moreover, the relative gene expression analysis revealed that the treatment of RXF393 cells raised the ratio of BAX/Bcl-2, indicating the percentage of cell apoptosis (Figure 9).

#### 2.2.7. Molecular Docking Studies

**Figure 10:**
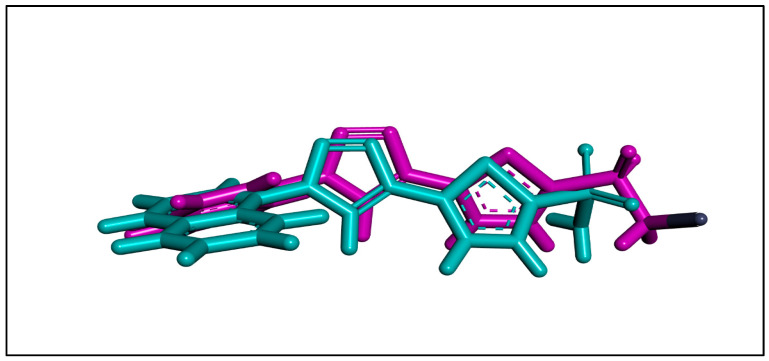
The superimposition of the redocked (violet color) and co-crystallized ligand (blue color) poses of CA IX active site (PDB 4FL4).

**Figure 11:**
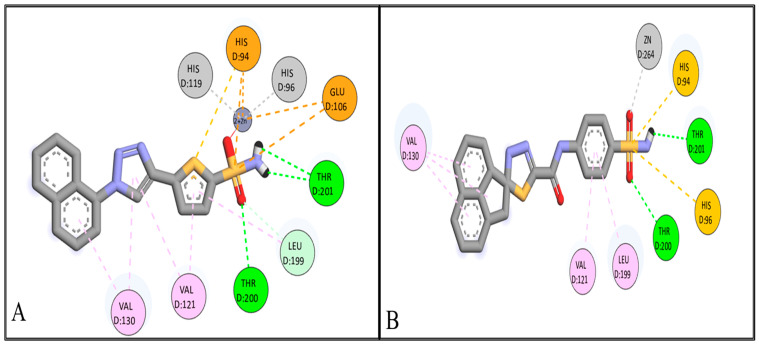
The 2D interactions of co-crystalized ligand IX (**A**) and the target compound **1** (**B**) in hCA IX active site (PDB 4FL4).

**Figure 12:**
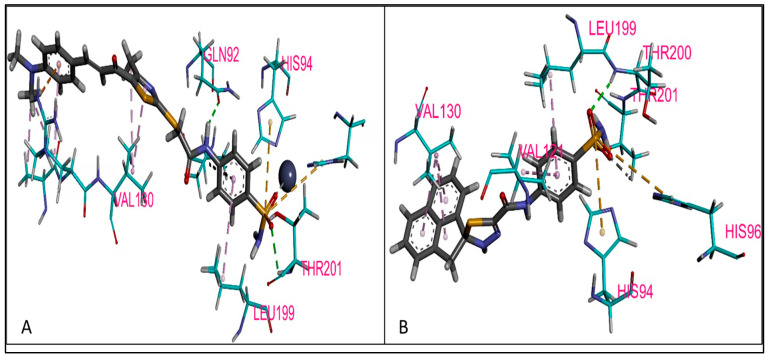
The 3D interactions of co-crystalized ligand IX (**A**) and the target compound **1** in hCA IX active site (**B**) (PDB 4FL4).

**Figure 13:**
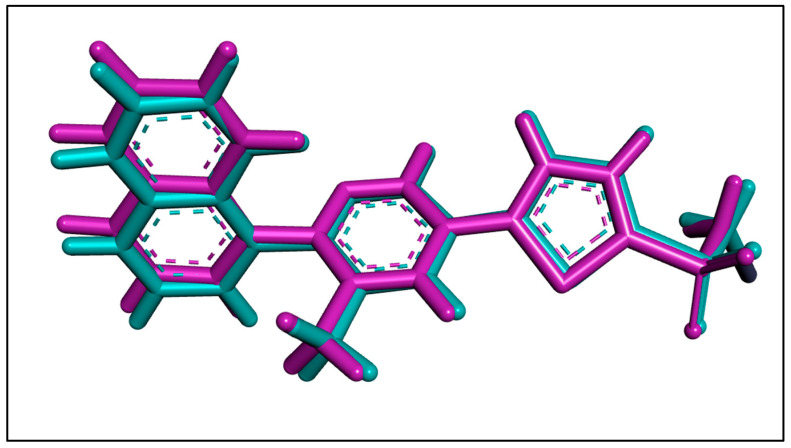
The superimposition of the redocked (violet color) and co-crystallized ligand (blue color) poses of CA XII active site (PDB 8CO3).

**Figure 14:**
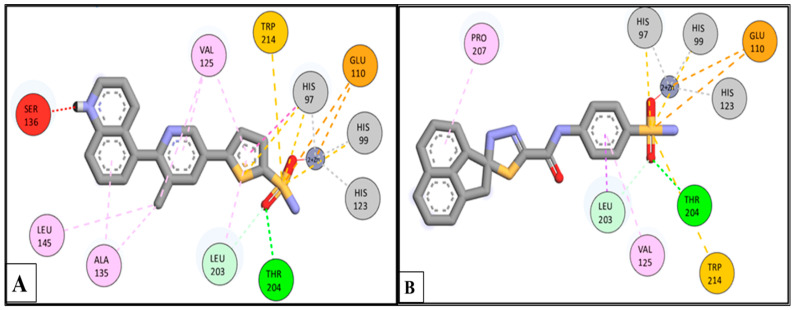
The 2D interactions of co-crystalized ligand XII (**A**) and the target compound **1** (**B**) in hCA XII active site (PDB 8CO3).

**Figure 15:**
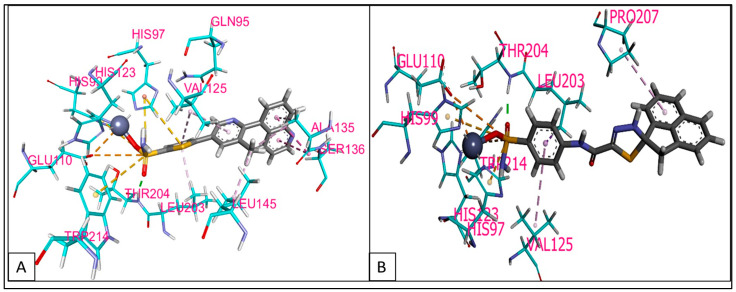
The 3D interactions of co-crystalized ligand XII (**A**) and the target compound **1** (**B**) in hCA XII active site (PDB 8CO3).

To explore the binding mode at the molecular level, the molecular docking of the target compound **1** was performed within the active site of hCA isoforms IX (PDB 4FL4) [22] and XII (8CO3) [41]. The docking parameters were validated for hCA IX by redocking the co-crystallized ligand (Figure 10) with low RMSD values, confirming their accuracy (Table 4 and Table 5). The target compound **1** was docked to investigate key interactions; the sulfonamide moiety functioned as a zinc-binding group, interacting with the Zn (II) ion via its amino groups HIS 94, HIS 96, and HIS 119. In the hCA IX active site, a hydrogen bond formed between the sulfamoyl S=O-NH group and THR 200 and THR 201. Additionally, the target compound **1** showed a pi–alkyl interaction with amino acid residue VAL 130 via the aromatic moiety. It is clear from Figure 11 and Figure 12 that the target compound **1** showed binding with the active site of CA IX and the co-crystallized ligand. Also, docking parameters were validated for hCA XII by redocking the co-crystallized ligand (Figure 13). Docking into hCA, XII revealed a hydrogen bond between the S=O group and the NH group of THR 204 for the target compound **1**, alongside π-π stacking between the phenyl ring of the benzene sulfonamide moiety and the LEU 203 and VAL 125 residues. Also, the aromatic tail of the target compound **1** showed pi–alkyl interaction with the PRO 207 amino acid residue (Figure 14 and Figure 15). Moreover, compound **1** showed good binding with the co-crystalized ligand with the active site of CA XII (PB: 8CO3). There is a great correlation between the docking data and the carbonic anhydrase inhibition assay results.

#### 2.2.8. Conclusions

This study successfully designed and synthesized a novel spiro-heterocyclic compound, *N-(4-(aminosulfinyl) phenyl)-2-oxo-2H3′H-spiro [acenaphthylene-12′-*[1,3,4] *thiadiazole]-5′-carboxamide*, as a selective inhibitor of tumor-associated carbonic anhydrase isoforms IX and XII. The compound demonstrated potent anticancer activity against various cancer cell lines, particularly renal RXF393, with superior efficacy compared to doxorubicin. Mechanistic studies revealed its ability to induce G1 phase cell cycle arrest and apoptosis in cancer cells, with lower toxicity toward normal cells, highlighting its therapeutic potential. Molecular docking confirmed strong binding affinities to the active sites of hCA IX and XII, further supporting its selectivity. The reduced toxicity against normal cells compared to doxorubicin underscores the compound’s safety profile. Overall, the target compound **1** shows great promise as a lead candidate for developing selective carbonic anhydrase inhibitors with potential applications in cancer therapy, warranting further investigation in preclinical and clinical settings.

## 3. Experimental Section

### 3.1. Chemistry

Reactions were monitored via TLC on aluminum pre-coated silica gel plates (2 cm × 5 cm, Kieselgel 60, Merk, Darmstadt, Germany) with a methylene chloride-to-methanol ratio of 19:1 as the eluent. Spots were detected using light from a UV lamp at a wavelength of 254 nm. Unadjusted melting points were determined utilizing an electrothermal melting point apparatus from Stuart Scientific Co. (Stone, UK). The Faculty of Science at Sohag University employs a Shimadzu 408 Spectrophotometer to obtain IR spectra in KBr discs. The Faculty of Science at Sohag University utilizes a Bruker AM NMR (400 MHz) spectrometer to acquire NMR spectra. All numerical values about NMR data are expressed in parts per million (ppm), with tetramethyl silane (TMS) serving as the reference standard. Elemental microanalyses for the synthesized compounds’ carbon, nitrogen, and hydrogen were performed using APCI as the ion source at the Regional Centre for Mycology and Biotechnology, Al-Azhar University, Cairo, Egypt.

#### 3.1.1. Synthesis of 2-Hydrazinyl-N-(4-sulfamoylphenyl)-2-thioxoacetamide

Synthesis was conducted via the reaction of 2-chloro-N-sulfamoylphenyl acetamide with morpholine and sulfur, followed by a reaction with hydrazine hydrate by a reported procedure [37].

Yellow crystals have been reported with the following: yield (82%); mp: 185–186 °C, reported as 186 °C [37]. ^1^H-NMR (DMSO-*d*_6_), δ ppm: 10.40 (s, 1H, NH amide), 7.86 (2H, d, *J*_H-H_ = 8.0 Hz, Ar-H), 7.80 (2H, d, *J*_H-H_ = 8.0 Hz, Ar-H), 7.28 (s, 2H, NH_2_SO_2_), and 3.81 (br, 3H, 3NH).

#### 3.1.2. Procedure of the Synthesis of Spiro-N-(4-sulfamoyl-phenyl)-1,3,4-thiadiazole-2-carboxamide Derivatives

Compounds **2**–**8** were synthesized as reported and confirmed by their melting point [37]. Meanwhile, the novel compound **1** was synthesized as mentioned below and characterized by spectroscopic tools and mass spectrometry.

General procedure of the synthesis of compound N-(4-(aminosulfinyl) phenyl)-2-oxo-2H, 3′H-spiro [acenaphthylene-1, 2′ [1,3,4] thiadiazole]-5′-carboxamide (compound **1**).

First, 1 mmol of acenaphthylene-1,2-dione was added to a solution of 2-hydrazinyl-N-(4-sulfamoylphenyl)-2-thioxoacetamide (1 mmol) in ethanol (15 mL), and the reaction mixture was then stirred at room temperature for about 3 h. The reaction was cooled, and the solid precipitate was collected by filtration, washed with ethanol, and dried [37].

Pale yellow crystal was reported with the following: yield: 0.381 g (87%); mp: 248–250 °C; IR (KBr) ύ (cm^−1^): 3346, 3271, 3178 (2NH, NH_2_), 3108 (CH-Ar), 2935, 2900 (CH-aliphatic), 1661 (C=O amide, st), and 1286 (S=O, st); ^1^H-NMR (400 MHz, DMSO-*d*_6_) δ ppm: 10.48 (s, 1H, NH amide), 10.18 (s, 1H, NH, thiadiazol), 7.93–7.29 (m, 7H, Ar), 7.01 (s, 2H, NH_2_); ^13^C-NMR (100 MHz, DMSO-*d*_6_) δ ppm: 182.60, 159.00, 140.80, 140.12, 135.03, 133.55, 132.62, 129.57, 128.26, 127.07, 120.47 and 86.19; Anal. Calcd for C_20_H_14_N_4_O_4_S_2_: C, 54.79; H, 3.22; N, 12.78; S, 14.62; Found: C, 54.67; H, 3.25; N, 12.83; S, 14.58; MS (APCl) calcd for C_20_H_13_N_4_O_4_S_2_ [M-H]^+^: 437.04, found: 437.00.

### 3.2. Biology

#### 3.2.1. *Screening of Anticancer Activity in the National Cancer Institute (NCI)*

The target compound’s anticancer efficacy was assessed at the National Cancer Institute (NCI), Bethesda, MD, USA, utilizing nine panels of 60 distinct cell lines sourced from nine human tumors typically accessible at the NCI library. The screening techniques are detailed on the NCI website (https://dtp.cancer.gov/, accessed on 1 November 2024) and we conducted them following NCI regulations [2]. For detailed information, see the Supplementary Materials.

#### 3.2.2. Evaluation of the IC_50_ of Compound **1** Against Melanoma LOX IMVI, Colon HT29, and Renal RXF393 Cancer Cell Lines in Addition to Normal Cell Line WI 38

The IC_50_ of the target compound against HT29, renal RXF393, melanoma LOX IMVI, and WI 38 cell lines was determined utilizing established MTT test procedures [2]. For detailed information, see the Supplementary Materials.

#### 3.2.3. Evaluation of Carbonic Anhydrase I, II, IV, and VII Inhibition

In vitro, the inhibition of cancer-associated carbonic anhydrase, hCA IX, and hCA XII of the target compound **1** was evaluated at the laboratory of the Egyptian company for the development of drugs, vaccines, and sera (VACSERA, Giza, Egypt) using the spectrophotometric technique that Pocker and Meany outlined [42,43].

#### 3.2.4. Cell Cycle Analysis

The impact of the target compound **1** on the cell cycle progression of the RXF393 cell line was assessed utilizing the Propidium Iodide Flow Cytometry Kit to quantify DNA content following established protocols [44]. For detailed information, see the Supplementary Materials.

#### 3.2.5. Apoptosis Determination Using Annexin V–Fluorescein Isothiocyanate/Propidium Iodide (FITC/PI) Staining

Cell apoptosis for the target compound **1** was analyzed using the Annexin V–FITC Apoptosis Detection Kit (Bio Vision Research Products, Miami, FL, USA) according to the reported protocols [45]. For detailed information, see the Supplementary Materials.

#### 3.2.6. RNA Isolation and Quantification

To begin, 5 × 10^5^ cells were cultured in triplicate on a 6-well plate. The cells were subsequently grown in DMEM medium under regulated circumstances of 5% CO_2_ and a temperature of 37 °C for 24 h. Then, the medium was substituted with DMEM containing the cells at its IC_50_ concentration, and the cells were then left for an additional 24 or 48 h prior to collection. Total RNA was isolated from both treated and untreated cells using TRizol^®^ (Invitrogen, Waltham, MA, USA) in accordance with the manufacturer’s instructions [46]. The Nano-Drop 1000 (Thermo Scientific, Waltham, MA, USA) was utilized to assess the quality and quantity of the extracted RNA [47].

#### 3.2.7. Analysis of Gene Expression by Real Time-PCR

In accordance with the manufacturer’s requirements, a high-capacity reverse transcriptase kit was utilized to reverse transcribe the mRNA pool using random hexamer primers. The reverse transcription process was executed as documented. Following a ten-minute cycle at 25 °C, a two-hour incubation at 37 °C was conducted, culminating in a five-minute incubation at 85 °C to ensure completion. The resultant cDNA was employed in a quantitative real-time polymerase chain reaction (qRT-PCR) utilizing the Maxima SYBR Green qPCR master mix (Thermo Scientific, USA). The procedure included an initial denaturation phase lasting 10 min at 95 °C, followed by 30 amplification cycles consisting of 15 s at 95 °C, 30 s at 60 °C, and 30 s at 72 °C. A final 10-min extension phase at 72 °C was included. Amplification was performed using a Step One Real-Time PCR System in accordance with the manufacturer’s instructions (Thermo Fisher, Waltham, MA, USA) [48,49]. All studies were performed in triplicate, utilizing the housekeeping gene Glyceraldehyde-3-phosphate dehydrogenase (GAPDH) as a reference in every experiment. The acquired qRT-PCR data were analyzed utilizing the comparative Ct method. The fold changes in treated cells were calculated by comparing them to untreated cells using the following formula: fold change = 2^−∆∆Ct^. Table 6 presents the primer sequences.

#### 3.2.8. Docking Studies

The crystal structures of h CA IX (PDB: 5FL4) and h CAXII (PDB: 4WW8) were retrieved from the protein data bank [40]. The proteins were prepared using AutoDock tools where the co-crystallized water molecules were removed then Kollman charges and polar hydrogens were added. The structure of compound **1** was drawn and optimized using Marvin Sketch V19.12 and Avogadro molecular editors [43]. The grid coordinates for h CAIX (PDB: 5FL4) and h CAXII were set to 15.13, −27.26, and 59.56 (h CAIX) and 25.63, 4.94, and 10.13 (h CA XII) for the x, y, and z axes, respectively, with grid dimensions of 20 × 20 × 20 for (h CA IX) and (hCA XII). AutoDock vina v1.2.0 was used for molecular docking, and the best docking poses were visualized using Discovery Studio Visualizer v24.1.0.23298 [50].

#### 3.2.9. Statistical Analysis

Data are presented as means ± SD. A one-way ANOVA, accompanied by the Bonferroni post hoc test for multiple comparisons, and a two-way ANOVA were conducted to evaluate the statistical significance of the differences utilizing the GraphPad Prism 9 software (GraphPad Software Inc., San Diego, CA, USA). Differences were considered significant when the *p*-value was below 0.05.

## 4. Discussion

Designing a novel anticancer agent via the inhibition of the only cancer-associated isoforms of hCA is a very important goal for medicinal chemists to avoid the side effects resulting from the inhibition of the cytosolic isoforms of CA, such as a metallic taste, loss of libido, fatigue, etc. There are various strategies for avoiding these side effects of unselectively inhibiting CA isoforms such as using local products like dorzolamide for the treatment of glaucoma, and a structural modification “tail approach” has emerged as a promising strategy involving changing the tail. The target compound was designed, synthesized, and evaluated as a novel anticancer agent via hCA cancer-associated inhibition in this context.

The target compound **1** was synthesized by replacing the para fluorophenyl moiety (tail moiety) with spiro acenaphthylene, aimed at greater selectivity towards CA IX and CA XII. The anticancer screening showed good cytotoxic effects against cancer cell lines such as K-562, HCT-116, HT29, LOX IMVI, IGROV1, RXF393, and MCF7 (NCI, USA) with a growth inhibition percentage range of 68.85–100%. The anti-proliferative activity of compound **1** is due to the inhibition of hCA, cell cycle arrest, and apoptosis induction. The inhibition of the cancer-associated isoforms CA IX and CA XII and cytosolic isoforms CA I and CA II by compound **1** were screened. Importantly, the results showed that compound **1** showed selectivity toward the cancer-associated isoform CA IX with an IC_50_ value of 0.477 μM, which is a very important target for designing novel anticancer activity via the inhibition of CA.

Moreover, the results of the carbonic anhydrase inhibition of compound **1** go ahead with the docking study within the active site of hCA isoforms IX (PDB 4FL4) and XII and (8CO3). Compound **1** was also docked to investigate key interactions; the sulfonamide moiety functioned as a zinc-binding group, interacting with the Zn (II) ion via its amino groups HIS 94, HIS 96, and HIS 119. In the hCA IX active site, a hydrogen bond formed between the sulfamoyl S=O-NH group and THR 200 and THR 201. Also, compound **1** showed pi–alkyl interaction with amino acid residue VAL 130 via the aromatic moiety. The timing of cell cycle withdrawal and differentiation is vital for appropriate growth and development, and it remains so throughout life. In contrast, failure to inhibit proliferation or the loss of differentiation can cause various disorders and are hallmarks of cancer cells. Here, we examine the molecular mechanisms that connect cell cycle arrest and the induction of apoptosis to the antineoplastic activity of the target compound, considering the potential implications for treating human cancer. The cytotoxic effect of compound **1** against RXF393 showed low IC_50_ with higher potency than the control, doxorubicin, used in this study.

To determine the effect of compound **1** on cell apoptosis, cells stained by Annexin V-FITC and PI after exposure to the compound **1**/RXF393 were analyzed by the flow cytometer. Cell line treatment with compound **1** showed a tangible effect on early and late apoptosis. The rate of apoptosis increased after treatment, confirming the results of the cytotoxic assay.

Apoptosis is initiated via two primary pathways: the extrinsic pathway, mediated by cell death receptors, and the intrinsic pathway, mediated by mitochondria. The extrinsic pathway leads to the activation of caspase-8 and caspase-9, respectively, which subsequently activate the downstream executioner caspase-3. Mitochondrial changes trigger the intrinsic apoptosis process, leading to the release of cytochrome c and a simultaneous decrease in mitochondrial transmembrane potential. Overall, the target compound **1** demonstrates significant potential as a lead candidate for the development of selective carbonic anhydrase inhibitors, with prospective applications in cancer therapy, necessitating additional exploration in preclinical and clinical contexts.

## Figures and Tables

**Table 1:** Inhibitory activity of the target compound **1** on hCA isoforms I, II, IX, and XII compared with acetazolamide, IC_50_ (μM).

Compound	hCA Inhibition IC_50_ (μM) ± SD				Selectivity Ratio			
	hCA I	hCA II	hCA IX	hCA XII	hCA I/ hCA IX	hCA II/ hCA IX	hCA I/ hCA XII	hCA II/ hCA XII
Compound **1**	7.353 ± 0.36	12.56 ± 0.74	0.477 ± 0.03	1.933 ± 0.11	15.41	26.33	3.80	6.50
Acetazolamide	0.367 ± 0.02	0.153 ± 0.01	0.105 ± 0.01	0.029 ± 0.001	3.49	1.56	12.56	5.27

**Table 2:** DNA content of the cell cycle of renal RXF393 cancer cell line after treatment with DMSO as a negative control, IC_50_ of doxorubicin as a positive control, and IC_50_ of the target compound **1** and their effect on the percentage of accumulation of cells at cell cycle different phases.

Compound	DNA Content		
	%G0-G1	%S	%G2/M
Compound **1**/RXF393	63.02	22.31	14.67
Doxorubicin/RXF393	72.18	16.81	11.01
DMSO/RXF393	51.95	28.54	19.51

**Table 3:** The apoptosis and necrosis assay of renal RXF393 cancer cell treated with IC_50_ concentration of the target compound **1** and doxorubicin, against positive control untreated cell as well as a negative control (RXF393-DMSO).

Compound	Apoptosis			Necrosis
	Total	Early	Late	
Compound **1**/RXF393	35.13	11.69	19.78	3.66
Doxorubicin/RXF393	39.44	12.64	17.36	9.02
DMSO/RXF393	1.76	0.42	0.18	1.16

**Table 4:** Molecular docking data for compound **1** and co-crystallized ligand CA IX against human carbonic anhydrase IX active site (PDB 4FL4).

Compound	RMSD	Binding Affinity (Kcal/mol)	Amino Acid Residues or DNA Nucleotide Bases	Types of Interaction
Co-crystallized ligand CA IX	1.33	−6.34	VAL 121	Pi–alkyl
			VAL 130	Pi–alkyl
			THR 200	H bond
			LEU 199	Pi–alkyl
			THR 201	H bond
			GLU 106	Attractive charge
			HIS 94	Attractive charge
			HIS 96	Metal interaction
			HIS 119	Metal interaction
Compound **1**	1.69	−6.74	VAL 121	Pi–alkyl
			VAL 130	Pi–alkyl
			LEU 199	Pi–alkyl
			HIS 94	Pi–sulfur
			HIS 96	Pi–sulfur
			THR 200	H bond
			THR 201	H bond
			ZN	Metal interaction

**Table 5:** Molecular docking data for the target compound **1** and coligand CA XII against human carbonic anhydrase XII active site (8CO3).

Compound	RMSD	Binding Affinity (Kcal/mol)	Amino Acid Residues or DNA Nucleotide Bases	Types of Interaction
Compound **1**	1.5098	−6.9990	Lue 203	Carbon hydrogen bond
			VAL 125	Pi–alkyl
			TRP 214	Pi–sulfur
			GLU 110	Attractive charge
			HIS 97	Metal interaction
			HIS 99	Metal interaction
			HIS 123	Metal interaction
			THR 204	H bond
			PRO 207	Pi–alkyl
Coligand XII	0.7712	−7.6961	SER 136	
			Lue 145	Pi–alkyl
			VAL125	Pi–alkyl
			HIS 97	Metal interaction
			HIS 99	Metal interaction
			HIS 123	Metal interaction
			THR 204	H bond
			ALA 135	Pi–alkyl
			GLU 110	Attractive charge
			TRP 214	Pi–sulfur
			LUE 203	

**Table 6:** Sequences of the primers.

Primer	Primer Sequence
*BAX*	Forward 5′-CTGCAGAGGATGATTGCCGC-3′ Reverse 5′-GGGCGTCCCAAAGTAGGAGA-3′
*Caspase 3*	Forward 5′-CTAGCGGATGGGTGCTATTGT-3′ Reverse 5′-AGAATGGGGGAAGAGGCAGG-3′
*Caspase 8*	Forward 5′-AGCCCTTGAGTTGGTCACTT-3′ Reverse 5′-CAGAAGTGGAACCTGTAGGCA-3′
*Caspase 9*	Forward 5′-TCAGGCCCCATATGATCGAG-3′ Reverse 5′-CAAGAGCACCGACATCACCA-3′
*P53*	Forward 5′-GGTGACACGCTTCCCTGGAT-3′ Reverse 5′-CATCCATTGCTTGGGACGGC-3′
*Bcl-2*	Forward 5′-CTGGTGGACAACATCGCCCT-3′ Reverse 5′-GCCGTACAGTTCCACAAAGGC-3′
*GAPDH*	Forward 5′-CGGGGCTCTCCAGAACATCAT-3′ Reverse 5′-GTCCACCACTGACACGTTGG-3′

## Data Availability

The original contributions presented in this study are included in the article; further inquiries can be directed to the corresponding author.

## Supplementary Materials

The following supporting information can be downloaded at: https://www.mdpi.com/article/10.3390/ijms26020863/s1.

## References

[1] Jemal A., Bray F., Center M.M., Ferlay J., Ward E., Forman D. (2011). Global cancer statistics. CA Cancer J. Clin..

[2] Aziz H.A., El-Saghier A.M., Badr M., Elsadek B.E., Abuo-Rahma G.E., Shoman M.E. (2024). Design, synthesis and mechanistic study of N-4-Piperazinyl Butyryl Thiazolidinedione derivatives of ciprofloxacin with Anticancer Activity via Topoisomerase I/II inhibition. Sci. Rep..

[3] Mohammed H.H., Abd El-Hafeez A.A., Ebeid K., Mekkawy A.I., Abourehab M.A., Wafa E.I., Alhaj-Suliman S.O., Salem A.K., Ghosh P., Abuo-Rahma G.E. (2022). New 1,2,3-triazole linked ciprofloxacin-chalcones induce DNA damage by inhibiting human topoisomerase I& II and tubulin polymerization. J. Enzym. Inhib. Med. Chem..

[4] Liao C., Liu X., Zhang C., Zhang Q. (2023). Tumor hypoxia: From basic knowledge to therapeutic implications. Semin. Cancer Biol..

[5] Chen Z., Han F., Du Y., Shi H., Zhou W. (2023). Hypoxic microenvironment in cancer: Molecular mechanisms and therapeutic interventions. Signal Transduct. Target. Ther..

[6] Ivan M., Fishel M.L., Tudoran O.M., Pollok K.E., Wu X., Smith P.J. (2022). Hypoxia signaling: Challenges and opportunities for cancer therapy. Semin. Cancer Biol..

[7] Paul S., Ghosh S., Kumar S. (2022). Tumor glycolysis, an essential sweet tooth of tumor cells. Semin. Cancer Biol..

[8] Mahon B.P., Bhatt A., Socorro L., Driscoll J.M., Okoh C., Lomelino C.L., Mboge M.Y., Kurian J.J., Tu C., Agbandje-McKenna M. (2016). The structure of carbonic anhydrase IX is adapted for low-pH catalysis. Biochemistry.

[9] Becker H.M. (2020). Carbonic anhydrase IX and acid transport in cancer. Br. J. Cancer.

[10] Queen A., Bhutto H.N., Yousuf M., Syed M.A., Hassan M.I. (2022). Carbonic anhydrase IX: A tumor acidification switch in heterogeneity and chemokine regulation. Semin. Cancer Biol..

[11] Kciuk M., Gielecińska A., Mujwar S., Mojzych M., Marciniak B., Drozda R., Kontek R. (2022). Targeting carbonic anhydrase IX and XII isoforms with small molecule inhibitors and monoclonal antibodies. J. Enzym. Inhib. Med. Chem..

[12] Chafe S.C., McDonald P.C., Saberi S., Nemirovsky O., Venkateswaran G., Burugu S., Gao D., Delaidelli A., Kyle A.H., Baker J.H. (2019). Targeting hypoxia-induced carbonic anhydrase IX enhances immune-checkpoint blockade locally and systemically. Cancer Immunol. Res..

[13] Nerella S.G., Thacker P.S., Arifuddin M., Supuran C.T. (2024). Tumor associated carbonic anhydrase inhibitors: Rational approaches, design strategies, structure activity relationship and mechanistic insights. Eur. J. Med. Chem. Rep..

[14] Al-Sanea M.M., Chilingaryan G., Abelyan N., Arakelov G., Sahakyan H., Arakelov V.G., Nazaryan K., Hussein S., Alazmi G.M., Alsharari H.E. (2021). Identification of non-classical hCA XII inhibitors using combination of computational approaches for drug design and discovery. Sci. Rep..

[15] Courcier J., de la Taille A., Nourieh M., Leguerney I., Lassau N., Ingels A. (2020). Carbonic Anhydrase IX in Renal Cell Carcinoma, Implications for Disease Management. Int. J. Mol. Sci..

[16] Singh P., Nerella S.G., Swain B., Angeli A., Ullah Q., Supuran C.T., Arifuddin M. (2024). Design, synthesis and in vitro evaluation of novel thiazole-coumarin hybrids as selective and potent human carbonic anhydrase IX and XII inhibitors. Int. J. Biol. Macromol..

[17] Tapera M., Kekeçmuhammed H., Tüzün B., Daştan S.D., Çelik M.S., Taslimi P., Dastan T., Topcu K.S., Cacan E., Şahin O. (2024). Novel 1,2,4-triazole-maleamic acid derivatives: Synthesis and evaluation as anticancer agents with carbonic anhydrase inhibitory activity. J. Mol. Struct..

[18] Hassan M.I., Shajee B., Waheed A., Ahmad F., Sly W.S. (2013). Structure, function and applications of carbonic anhydrase isozymes. Bioorg. Med. Chem..

[19] Begines P., Bonardi A., Nocentini A., Gratteri P., Giovannuzzi S., Ronca R., Tavani C., Massardi M.L., López Ó., Supuran C.T. (2023). Design and synthesis of sulfonamides incorporating a biotin moiety: Carbonic anhydrase inhibitory effects, antiproliferative activity and molecular modeling studies. Bioorg. Med. Chem..

[20] Abdelhakeem M.M., Morcoss M.M., Hanna D.A., Lamie P.F. (2024). Design, synthesis and in silico insights of novel 1,2,3-triazole benzenesulfonamide derivatives as potential carbonic anhydrase IX and XII inhibitors with promising anticancer activity. Bioorg. Chem..

[21] Buravchenko G.I., Scherbakov A.M., Krymov S.K., Salnikova D.I., Zatonsky G.V., Schols D., Vullo D., Supuran C.T., Shchekotikhin A.E. (2024). Synthesis and evaluation of sulfonamide derivatives of quinoxaline 1,4-dioxides as carbonic anhydrase inhibitors. RSC Adv..

[22] Hefny S.M., El-Moselhy T.F., El-Din N., Ammara A., Angeli A., Ferraroni M., El-Dessouki A.M., Shaldam M.A., Yahya G., Al-Karmalawy A.A. (2024). A new framework for novel analogues of pazopanib as potent and selective human carbonic anhydrase inhibitors: Design, repurposing rational, synthesis, crystallographic, in vivo and in vitro biological assessments. Eur. J. Med. Chem..

[23] Krymov S.K., Scherbakov A.M., Salnikova D.I., Sorokin D.V., Dezhenkova L.G., Ivanov I.V., Vullo D., De Luca V., Capasso C., Supuran C.T. (2022). Synthesis, biological evaluation, and in silico studies of potential activators of apoptosis and carbonic anhydrase inhibitors on isatin-5-sulfonamide scaffold. Eur. J. Med. Chem..

[24] Higazy S., Samir N., El-Khouly A., Giovannuzzi S., Begines P., Gaber H.M., Supuran C.T., Abouzid K.A. (2024). Identification of thienopyrimidine derivatives tethered with sulfonamide and other moieties as carbonic anhydrase inhibitors: Design, synthesis and anti-proliferative activity. Bioorg. Chem..

[25] Ibrahim S.A., Al-Mhyawi S.R., Atlam F.M. (2023). New imidazole-2-ones and their 2-thione analogues as anticancer agents and CAIX inhibitors: Synthesis, in silico ADME and molecular modeling studies. Bioorg. Chem..

[26] Akocak S., Güzel-Akdemir Ö., Sanku R.K., Russom S.S., Iorga B.I., Supuran C.T., Ilies M.A. (2020). Pyridinium derivatives of 3-aminobenzenesulfonamide are nanomolar-potent inhibitors of tumor-expressed carbonic anhydrase isozymes CA IX and CA XII. Bioorg. Chem..

[27] Kumar S., Rulhania S., Jaswal S., Monga V. (2021). Recent advances in the medicinal chemistry of carbonic anhydrase inhibitors. Eur. J. Med. Chem..

[28] Tykvart J., Navrátil V., Kugler M., Šácha P., Schimer J., Hlaváčková A., Tenora L., Zemanová J., Dejmek M., Král V. (2020). Identification of novel carbonic anhydrase IX inhibitors using high-throughput screening of pooled compound libraries by DNA-linked inhibitor antibody assay (DIANA). Slas Discov. Adv. Sci. Drug Discov..

[29] Abdel-Mohsen H.T., El Kerdawy A.M., Omar M.A., Petreni A., Allam R.M., El Diwani H.I., Supuran C.T. (2022). Application of the dual-tail approach for the design and synthesis of novel Thiopyrimidine–Benzenesulfonamide hybrids as selective carbonic anhydrase inhibitors. Eur. J. Med. Chem..

[30] Tawfik H.O., Petreni A., Supuran C.T., El-Hamamsy M.H. (2022). Discovery of new carbonic anhydrase IX inhibitors as anticancer agents by toning the hydrophobic and hydrophilic rims of the active site to encounter the dual-tail approach. Eur. J. Med. Chem..

[31] Krasavin M., Kalinin S., Sharonova T., Supuran C.T. (2020). Inhibitory activity against carbonic anhydrase IX and XII as a candidate selection criterion in the development of new anticancer agents. J. Enzym. Inhib. Med. Chem..

[32] Carta F., Vullo D., Angeli A. (2021). Design, synthesis, and biological evaluation of selective hCA IX inhibitors. pH-Interfering Agents as Chemosensitizers in Cancer Therapy.

[33] Kumar A., Siwach K., Supuran C.T., Sharma P.K. (2022). A decade of tail-approach based design of selective as well as potent tumor associated carbonic anhydrase inhibitors. Bioorg. Chem..

[34] Bondock S., Albarqi T., Abboud M., Nasr T., Mohamed N.M., Abdou M.M. (2023). Tail-approach based design, synthesis, and cytotoxic evaluation of novel disubstituted and trisubstituted 1,3-thiazole benzenesulfonamide derivatives with suggested carbonic anhydrase IX inhibition mechanism. RSC Adv..

[35] Vats L., Siwach K., Angeli A., Bikal P., Bhardwaj J.K., Supuran C.T., Sharma P.K. (2023). Tail approach synthesis of triazolylthiazolotriazole bearing benzenesulfonamides as carbonic anhydrase inhibitors capable of inducing apoptosis. Arch. Pharm..

[36] Abo-Ashour M.F., Eldehna W.M., Nocentini A., Ibrahim H.S., Bua S., Abdel-Aziz H.A., Abou-Seri S.M., Supuran C.T. (2019). Novel synthesized SLC-0111 thiazole and thiadiazole analogues: Determination of their carbonic anhydrase inhibitory activity and molecular modeling studies. Bioorg. Chem..

[37] El-Saghier A.M., Enaili S.S., Abdou A., Kadry A.M. (2024). An efficient eco-friendly, simple, and green synthesis of some new spiro-N-(4-sulfamoyl-phenyl)-1,3,4-thiadiazole-2-carboxamide derivatives as potential inhibitors of SARS-CoV-2 proteases: Drug-likeness, pharmacophore, molecular docking, and DFT exploration. Mol. Divers..

[38] Badran M.M., Abbas S.H., Fujita M., Abdel-Aziz M. (2023). Harnessing pyrimidine as a building block for histone deacetylase inhibitors. Arch. Pharm..

[39] Kaina B. (2003). DNA damage-triggered apoptosis: Critical role of DNA repair, double-strand breaks, cell proliferation and signaling. Biochem. Pharmacol..

[40] Elmore S. (2007). Apoptosis: A review of programmed cell death. Toxicol. Pathol..

[41] TSupuran C., Di Fiore A., Alterio V., Maria Montib S., De Simone G. (2010). Recent advances in structural studies of the carbonic anhydrase family: The crystal structure of human CA IX and CA XIII. Curr. Pharm. Des..

[42] Pocker Y., Meany J.E. (1967). The catalytic versatility of erythrocyte carbonic anhydrase. II. Kinetic studies of the enzyme-catalyzed hydration of pyridine aldehydes. Biochemistry.

[43] Ahmed R.F., Mahmoud W.R., Abdelgawad N.M., Fouad M.A., Said M.F. (2023). Exploring novel anticancer pyrazole benzenesulfonamides featuring tail approach strategy as carbonic anhydrase inhibitors. Eur. J. Med. Chem..

[44] Saied S., Shaldam M., Elbadawi M.M., Giovannuzzi S., Nocentini A., Almahli H., Salem R., Ibrahim T.M., Supuran C.T., Eldehna W.M. (2023). Discovery of indolinone-bearing benzenesulfonamides as new dual carbonic anhydrase and VEGFR-2 inhibitors possessing anticancer and pro-apoptotic properties. Eur. J. Med. Chem..

[45] Al-Hakkani M.F., Ahmed N., Abbas A.A., Hassan M.H., Aziz H.A., Elshamsy A.M., Khalifa H.O., Abdelshakour M.A., Saddik M.S., Elsayed M.M. (2023). Synthesis, Physicochemical Characterization using a Facile Validated HPLC Quantitation Analysis Method of 4-Chloro-phenylcarbamoyl-methyl Ciprofloxacin and Its Biological Investigations. Int. J. Mol. Sci..

[46] Hummon A.B., Lim S.R., Difilippantonio M.J., Ried T. (2007). Isolation and solubilization of proteins after TRIzol^®^ extraction of RNA and DNA from patient material following prolonged storage. Biotechniques.

[47] Boesenberg-Smith K.A., Pessarakli M.M., Wolk D.M. (2012). Assessment of DNA yield and purity: An overlooked detail of PCR troubleshooting. Clin. Microbiol. Newsl..

[48] Longo M.C., Berninger M.S., Hartley J.L. (1990). Use of uracil DNA glycosylase to control carry-over contamination in polymerase chain reactions. Gene.

[49] Almasmoum H. (2021). Journal of Umm Al-Qura University for Medical Sciences. J. Umm Al-Qura Univ. Med. Sci..

[50] Ali D.M., Aziz H.A., Bräse S., Al Bahir A., Alkhammash A., Abuo-Rahma G.E., Elshamsy A.M., Hashem H., Abdelmagid W.M. (2024). Unveiling the Anticancer Potential of a New Ciprofloxacin-Chalcone Hybrid as an Inhibitor of Topoisomerases I & II and Apoptotic Inducer. Molecules.

