# Supplementary material for: Design, Synthesis, Anticancer Screening, and Mechanistic Study of Spiro-N-(4-sulfamoyl-phenyl)-1,3,4-thiadiazole-2-carboxamide Derivatives
Source: Int J Mol Sci. 2025 Jan 20;26(2):863. doi: 10.3390/ijms26020863 (PMC11766273; doi:10.3390/ijms26020863)
Supplement: Supplementary file 1 [file ijms-26-00863-s001.zip › ijms-3342167-supplementary.pdf]

Supporting Information

Design, Synthesis, Anticancer Screening, and Mechanistic Study of Spiro-N-(4-sulfamoyl-phenyl)-1,3,4-thiadiazole-2-carboxamide Derivatives

Ahmed M. El-Saghier [1,*], Hamada Hashem [2], Sherif A. Maher [3], Souhaila S. Enaili [4], Abdullah Alkhammash [5], Stefan Bräse [6,*] and Hossameldin A. Aziz [7]

[1] Department of Chemistry, Faculty of Science, Sohag University, Sohag 82524, Egypt
[2] Department of Pharmaceutical Chemistry, Faculty of Pharmacy, Sohag University, Sohag 82524, Egypt; hamada.hashem@pharm.sohag.edu.eg
[3] Department of Biochemistry, Faculty of Pharmacy, New Valley University, New Valley 72511, Egypt; sherif.ali87@pha.nvu.edu.eg
[4] Department of Chemistry, Faculty of Science, University of Zawia, Az Zawiyah 16418, Libya; s.enaili@zu.edu.ly
[5] Department of Pharmacology, College of Pharmacy, Shaqra University, Shaqra 11961, Saudi Arabia; alkhammash@su.edu.sa
[6] Institute for Biological and Chemical System, Karlsruhe Institute of Technology, 76131 Karlsruhe, Germany
[7] Department of Pharmaceutical Chemistry, Faculty of Pharmacy, New Valley University, New Valley 72511, Egypt; hossamaziz85@pha.nvu.edu.eg
* Correspondence: el.saghier@science.sohag.edu.eg (A.M.E.-S.); stefan.braese@kit.edu (S.B.)

Screening of anti-cancer activity in the National Cancer Institute (NCI)

The target compound anti-cancer efficacy was assessed at the National Cancer Institute (NCI), USA, utilizing nine panels of 60 distinct cell lines sourced from nine human tumors typically accessible at the NCI library. The screening techniques were detailed on the NCI website (http://www.dtp.nci.nih.gov) and conducted following NCI regulations. Briefly, a primary anticancer assay was performed at approximately sixty human tumor cell line panels derived from nine neoplastic diseases following the protocol of the Drug Evaluation Branch, National Cancer Institute, Bethesda. Tested compounds were added to the culture at a single concentration (10^{-5} M), and the cultures were incubated for 48 h. End-point determinations were made with a protein-binding dye, sulforhodamine B (SRB). Results for each tested compound were reported as the percent of growth of the treated cells when compared to the untreated control cells. The percentage growth was evaluated spectrophotometrically versus controls not treated with test agents. All final compounds that showed significant cell growth inhibition in the One-Dose Screen were evaluated against the 60-cell panel at five different concentrations by solubilizing the drug in dimethyl sulfoxide. After drug addition, cells were incubated at 37 ◦C, 5 % CO2, 95 % air, and 100 % relative humidity for 48 h, then stained by SRB, and the absorbance was evaluated spectrophotometrically by using an

automated plate. The growth percentage was calculated at different drug concentration levels and at different *times.*

Evaluation of the IC_{50} of the target compound against melanoma LOX IMVI, colon HT29, and renal RXF393 cancer cell lines in addition to normal cell line WI 38.

The IC_{50} of the target compound against HT29, renal RXF393, melanoma LOX IMVI, and WI 38 cell lines was determined utilizing established MTT test procedures.
All cell lines utilized were acquired from the Vacsera Cell Culture Library, Tissue Culture Unit, Cairo, Egypt, with ATCC provenance. Tissue Culture Laboratory. The concentrations that inhibited 50% of cell growth were tested three times, and the mean was computed. Cells were grown in DMEM (Invitrogen/Life Technologies) augmented with 10% FBS (Hyclone), 10 mg/ml of insulin (Sigma), and 1% penicillin-streptomycin. Seed cells (density 1.2–1.8×10,000 cells/well) in 100 mL of complete growth medium and 100 mL of the tested chemical per well in a 96-well plate for 24 h before the MTT testing. In a standard experiment, 100 mL of serially diluted sterile testing substances were introduced to achieve final concentrations ranging from 0.01 to 100 mM, utilizing growth media as a negative control. Following 24 h of culture incubation, the supernatants were discarded. The LOX IMVI, HT29, and RXF393 cell lines were subjected to trypsinization and rinsed with calcium/magnesium-free PBS (pH 7.2). We transferred cultures from the incubator to a laminar flow hood or sterile workspace. Utilize cells in the logarithmic growth phase, ensuring the final cell count does not surpass 106 cells/cm^2. Each test must incorporate a blank, providing complete media devoid of cells. Reconstitute each vial of MTT [M-5655] using 3 ml of medium or balanced salt solution devoid of phenol red and serum. Incorporate reconstituted MTT at a volume constituting 10% of the culture medium—re-incubate cultures for 2 to 4 h, contingent upon cell type and optimal cell density. Upon completion of the incubation period, the cultures from the incubator are extracted, and the resultant formazan crystals are dissolved by adding an equivalent volume of MTT Solubilization Solution [M-8910] to that of the original culture media. Absorbance was spectrophotometrically measured at a wavelength of 570 nm. Quantify the background absorbance of multi-well plates at 690 nm and deduct this value from the data at 450 nm. Data from all experiments were documented, and the viability percentage of cells was computed.

Cell cycle analysis

The impact of the target compound on the cell cycle progression of the RXF393 cell line was assessed utilizing the Propidium Iodide Flow Cytometry Kit to quantify DNA content following established protocols. For detailed information, see Appendix A in the Supplementary Materials.

RXF393 cells were treated with the IC_{50} concentration of the target compound (7.01±0.39). The effect of the target compound on apoptosis induction in the RXF393 cell line was evaluated against conventional doxorubicin and untreated cells, serving as positive and negative controls, respectively. Following the manufacturer's guidelines, the Annexin V-FITC Apoptosis Detection Kit (Bio Vision Research Products, USA) was employed to assess cellular apoptosis. In summary, 500 µL of 1X Binding buffer was utilized to resuspend 1-5 x 105 cells collected via centrifugation. Propidium iodide (PI, 50 mg/ml) and Annexin V-FITC, delivered in 5 µL, were also included. The cells were initially incubated for 5 minutes at ambient temperature in darkness before analysis via the Annexin V-FITC binding flow cytometric method (Ex=488 nm; Em=530 nm), utilizing a FITC signal detector (commonly FL1) and PI staining with a phycoerythrin emission signal detector (typically FL2). Before exposing adherent cells to Annexin V-FITC, we carefully trypsinized them and performed a single wash in serum-containing media (A.3-5). Response curve equation with graph Pad Prism 7 software (GraphPad Software Inc., San Diego, CA, USA).

Apoptosis Determination Using Annexin V-Fluorescein isothiocyanate/Propidium iodide (FITC/PI) Staining

Annexin V-FITC Apoptosis Detection Kit (Catalog #: K101-25, Bio Vision Research Products, 980 Linda Vista Avenue, Mountain View, CA 94043, USA) The phenomenon is predicated on the finding that shortly after the onset of apoptosis, cells relocate the membrane phosphatidylserine (PS) from the inner aspect of the plasma membrane to the extracellular surface. Upon reaching the cell surface, phosphatidylserine (PS) is readily identifiable through staining with a fluorescent compound of Annexin V, a protein exhibiting a strong affinity for PS. The one-step staining technique requires merely 10 minutes, followed by detection assessed using flow cytometry. The kit can distinguish between apoptosis and necrosis during Annexin V-FITC and PI labeling. Consequently, early apoptotic cells are characterized as Annexin V-positive and PI-negative, while late apoptotic cells are both Annexin V-positive and PI-positive. Utilizing the Apoptosis detection kit, cells (5×10^5) were harvested in triplicate in Dulbecco's Modified Eagle Medium (DMEM) and incubated at 37 °C in a 5% CO_2 environment for 24 h to facilitate attachment. The media was subsequently substituted with DMEM containing the tested chemical (IC_{50}), and the cells were cultured for 48 hours before harvesting. Cells were washed with cold PBS and subsequently resuspended in the binding buffer. Thereafter, annexin V and propidium iodide were

applied to the cells at 4 °C for 30 minutes without light for cell staining. The FACS Calibur flow cytometer (Becton Dickinson, Franklin Lakes, NJ, USA) was employed to analyze a minimum of 10^4 cells. As previously described, dot plots were created, and the total apoptosis fraction was evaluated.

RNA Isolation and Quantification

5×10^5 cells have been cultivated in triplicate on a 6-well plate. The cells were then cultured in DMEM medium under controlled conditions of 5% CO_2 and 37 °C temperature for 24 h. Following that, the medium was replaced with DMEM containing the cells at its IC_{50} concentration, and the cells were further maintained for another 24 or 48 h before being collected. For total RNA isolation from both the treated and untreated cells, TRizol® (Invitrogen, USA) was used according to the manufacturer's guidelines. Nano-Drop 1000 (Thermo Scientific, Waltham, MA, USA) was employed to determine the quality and amount of the obtained RNA.

1. Chemistry

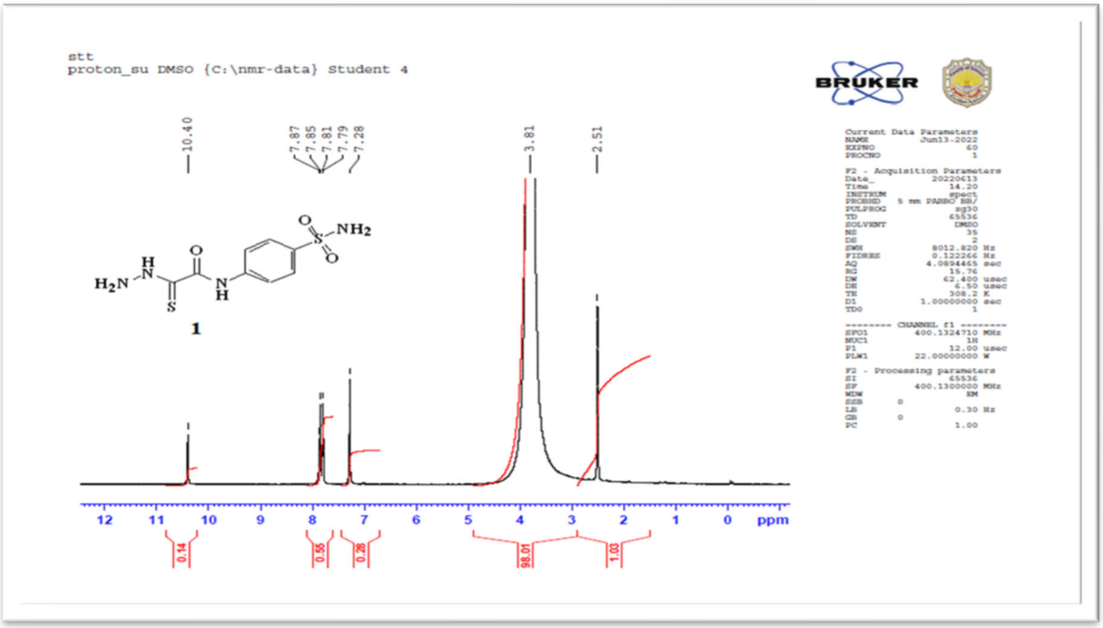

Figure S1. ^{1}HNMR spectrum of the compound 1

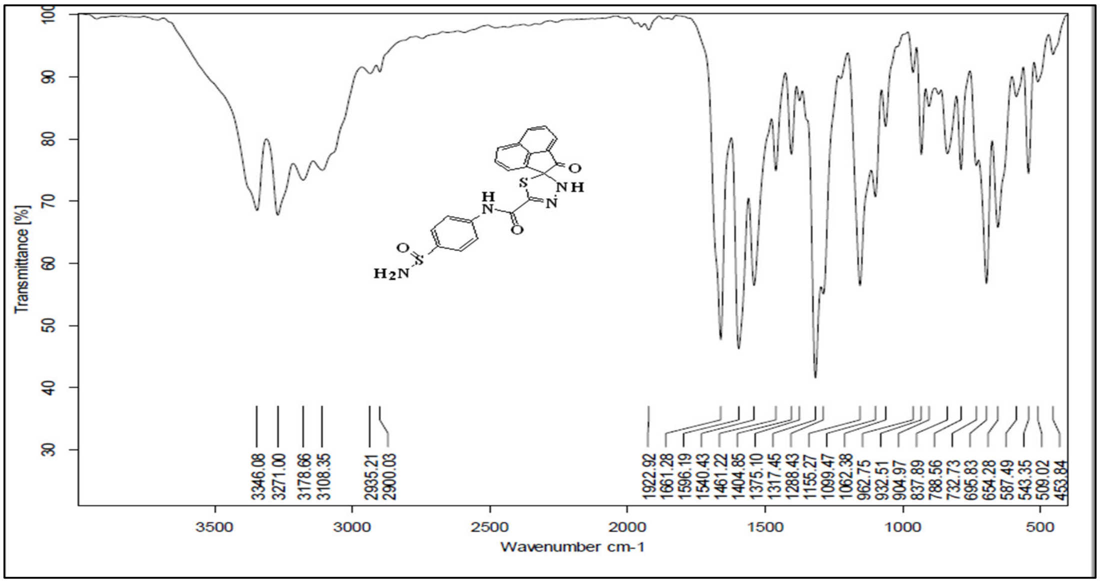

Figure S2. IR spectrum of the target compound

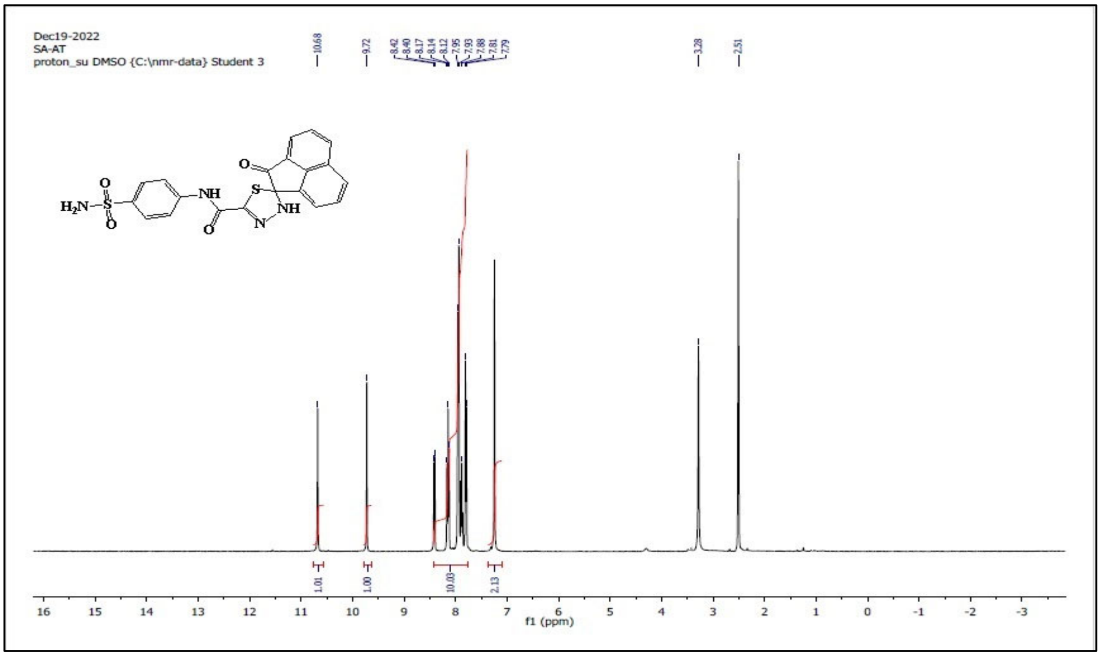

Figure S3. ^{1}HNMR spectrum of the target compound

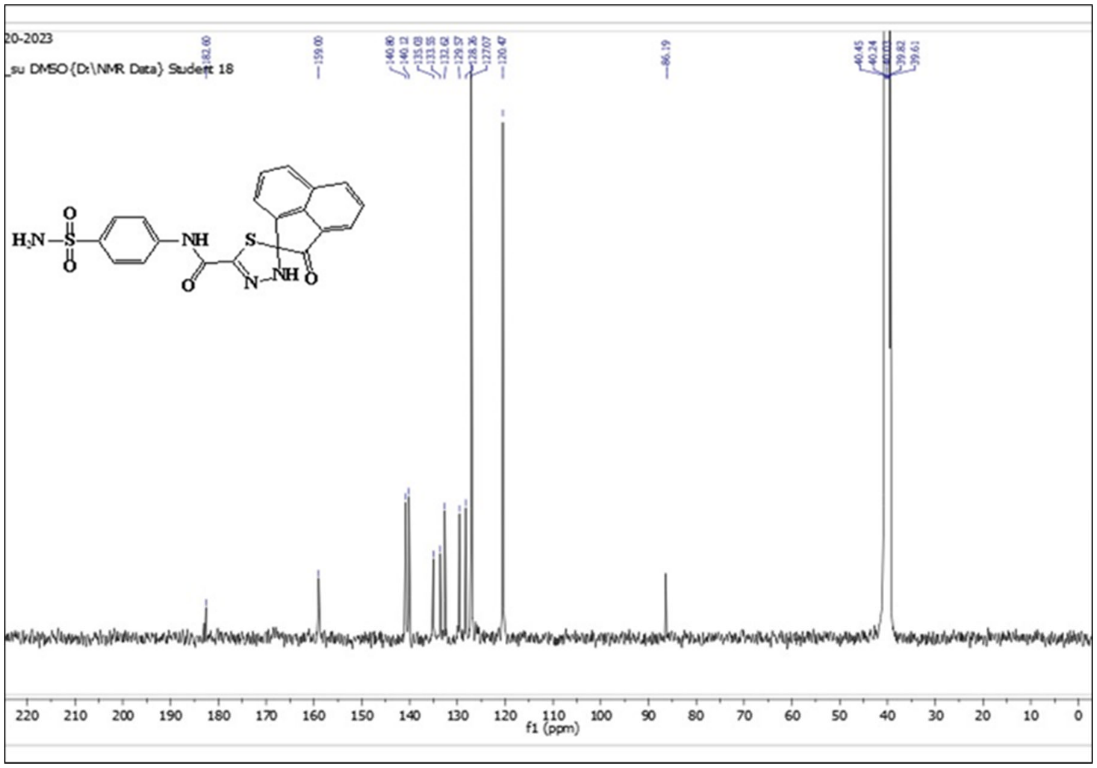

Figure S4. [13] CNMR spectrum of the target compound (100 MHz, DMSO-d_6)

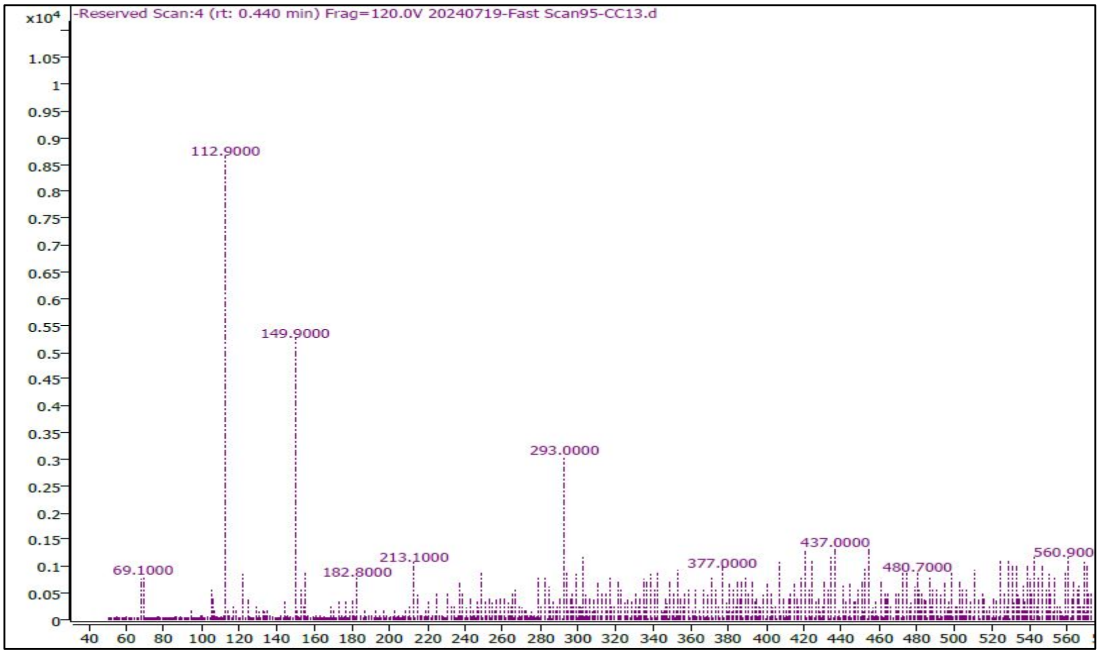

Figure S5. Mass spectrum of the target compound

2. Biology

2.1. One dose anticancer screening of the target compounds 1-8 (NCI, USA, at concentration of 10 μM)

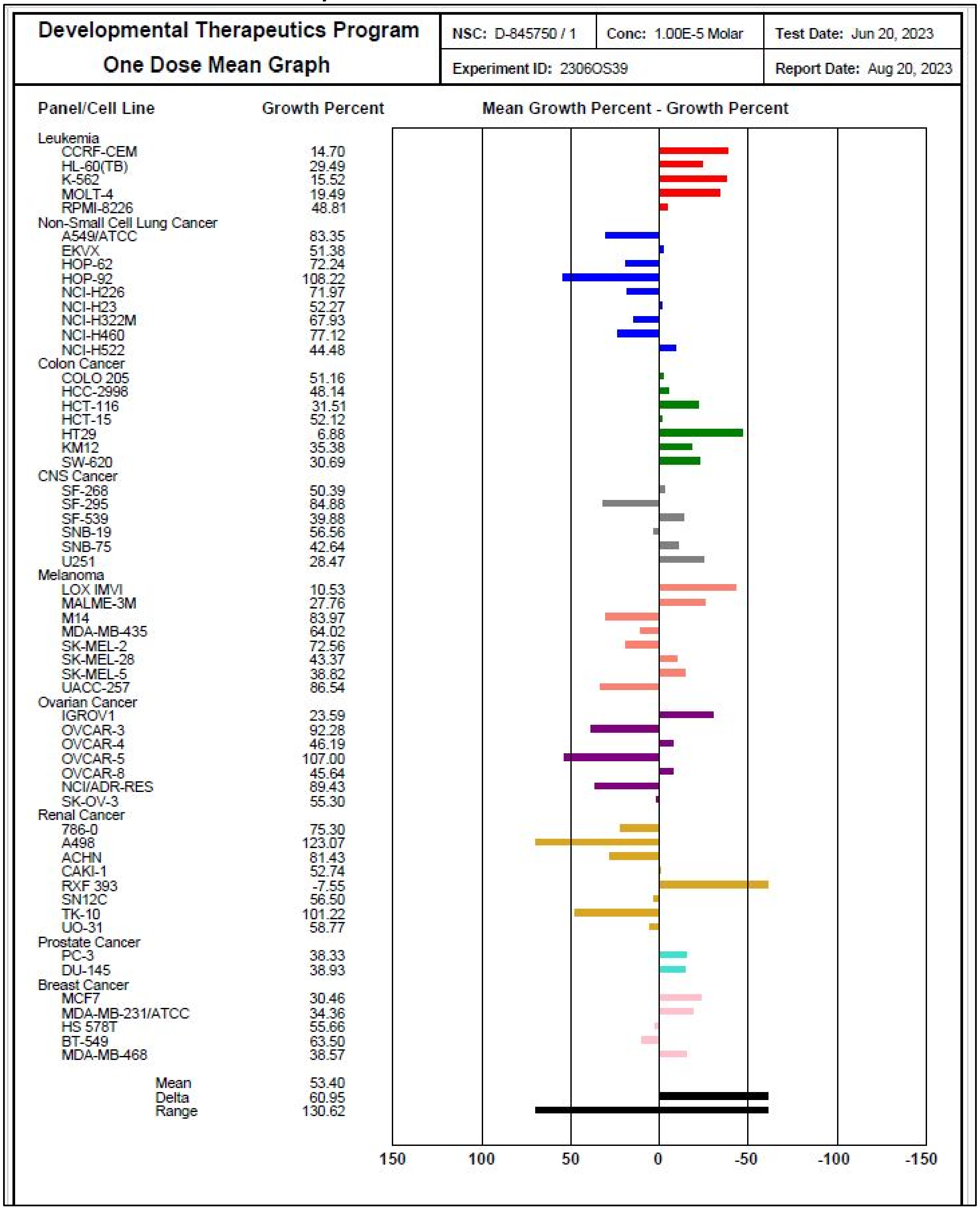

Figure S6. One dose anticancer screening of compound 1 (NCI, USA)

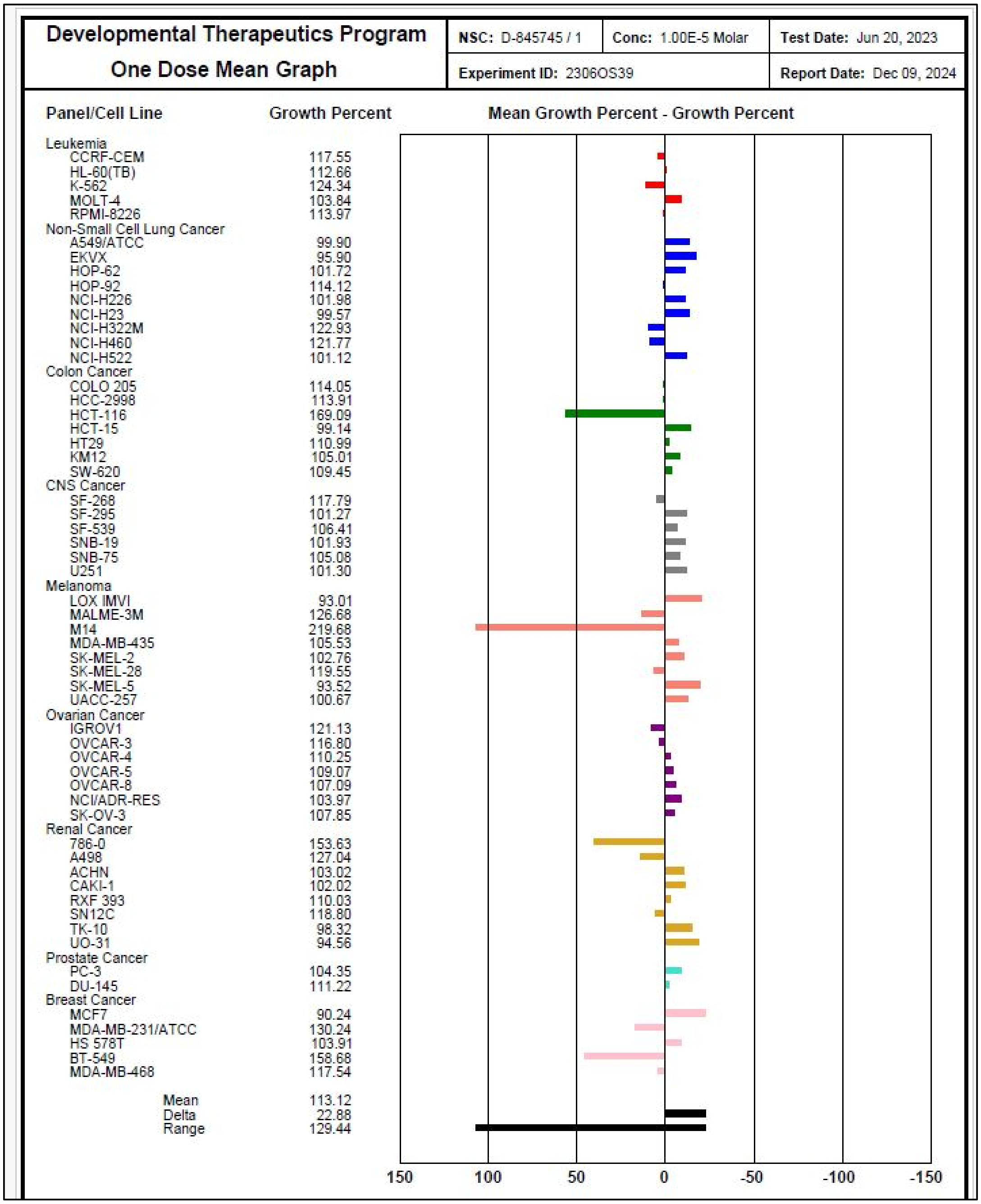

Figure S7. One dose anticancer screening of compound 2 (NCI, USA)

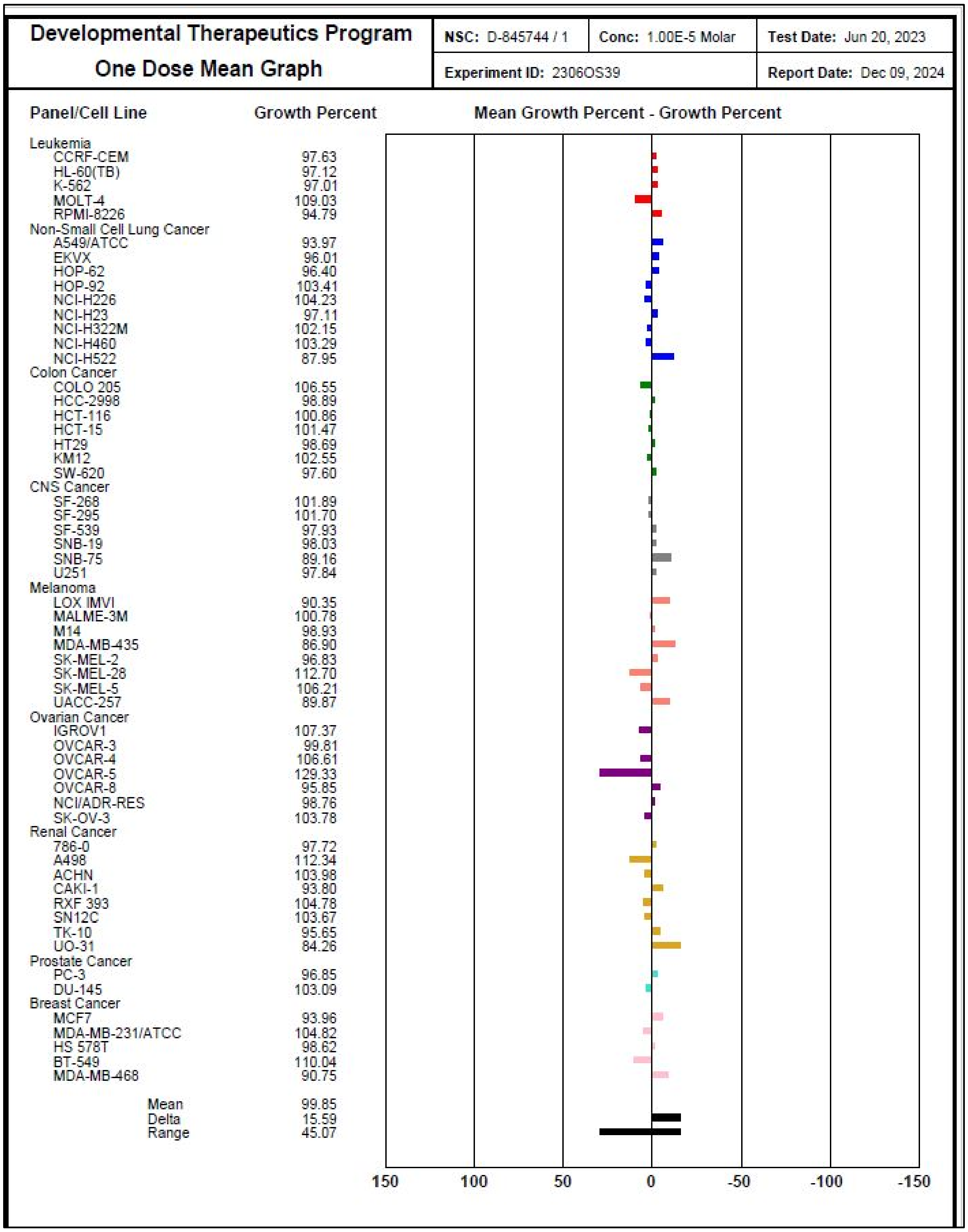

Figure S8. One dose anticancer screening of compound 3 (NCI, USA)

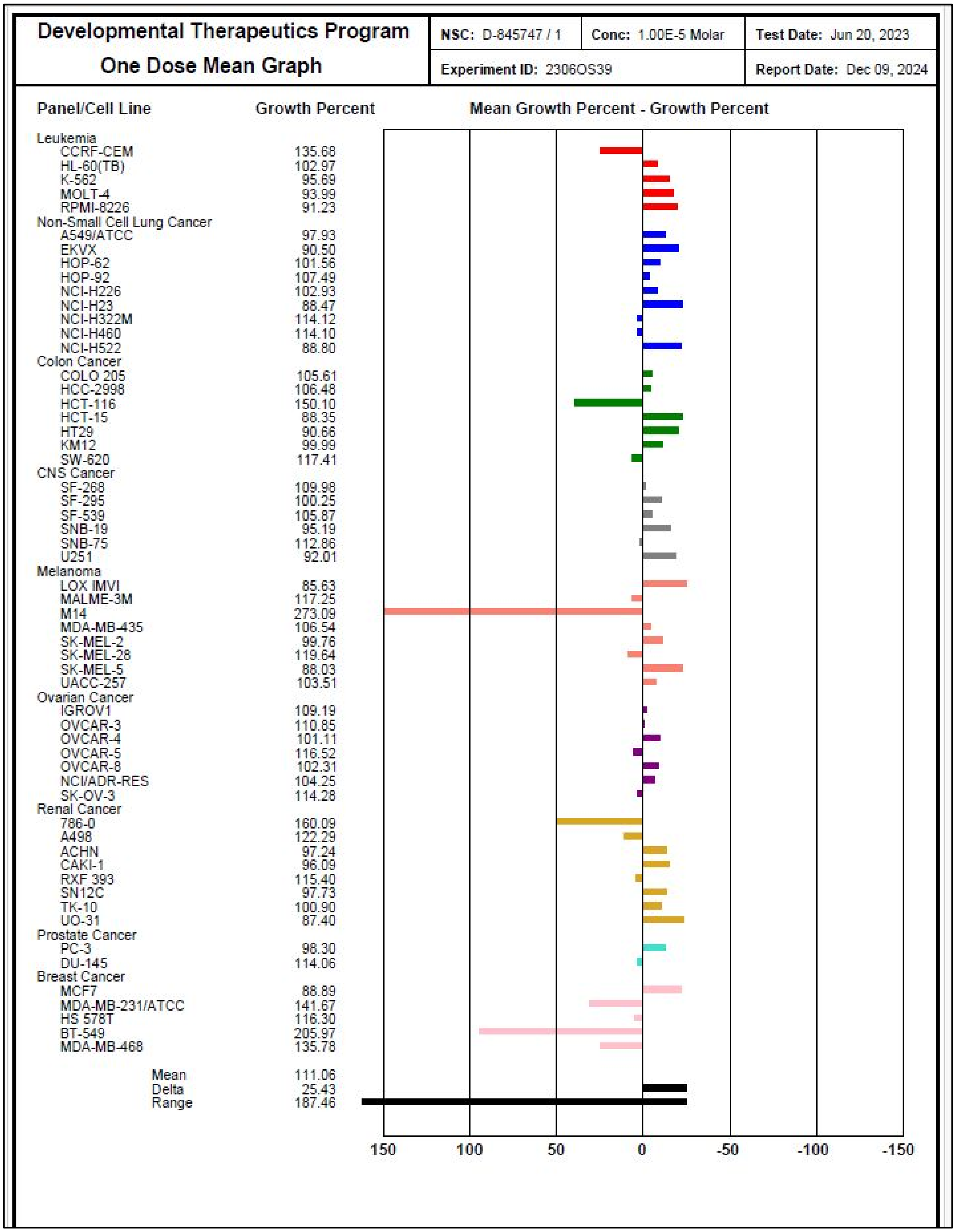

Figure S9. One dose anticancer screening of compound 4 (NCI, USA)

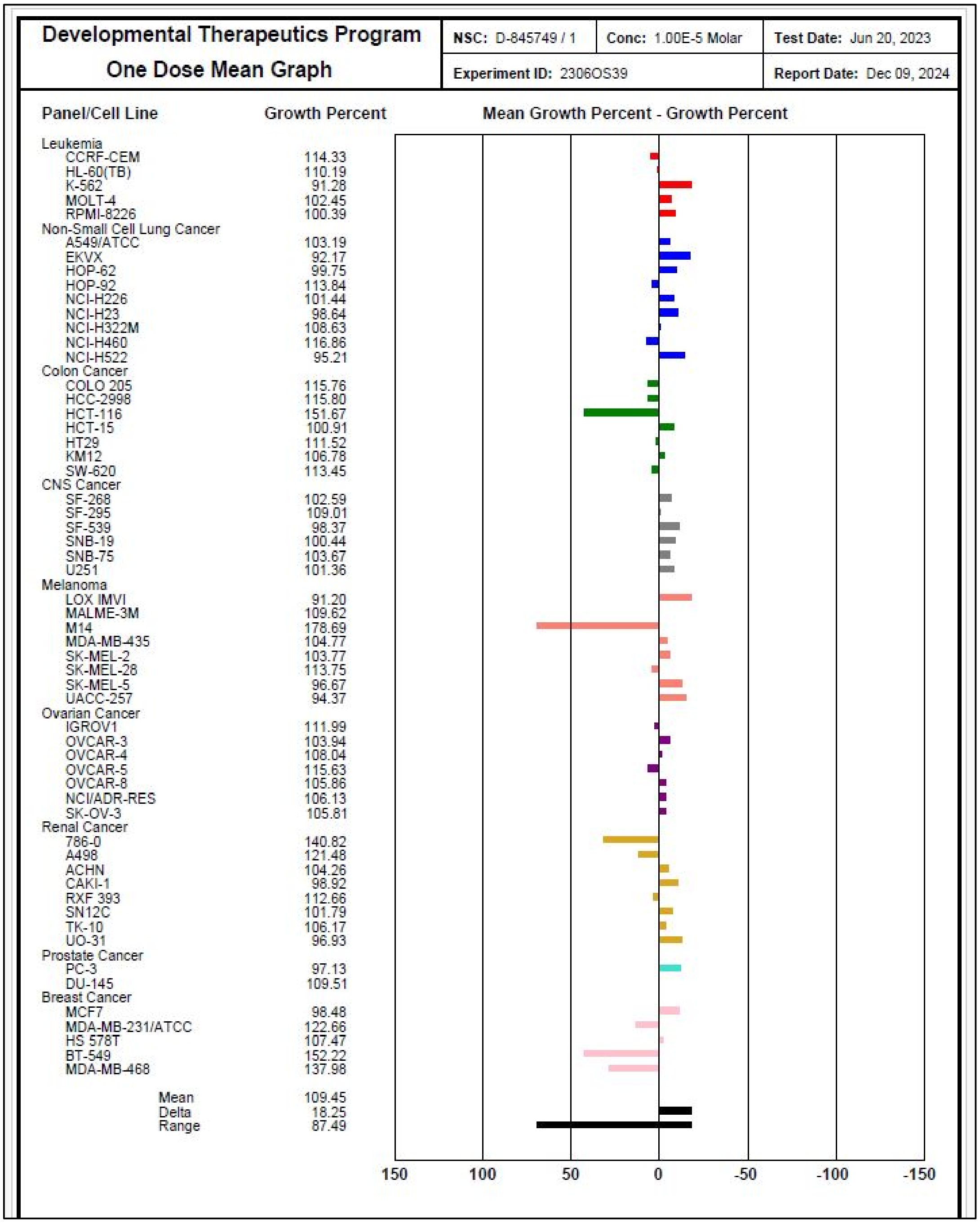

Figure S10. One dose anticancer screening of compound 5 (NCI, USA)

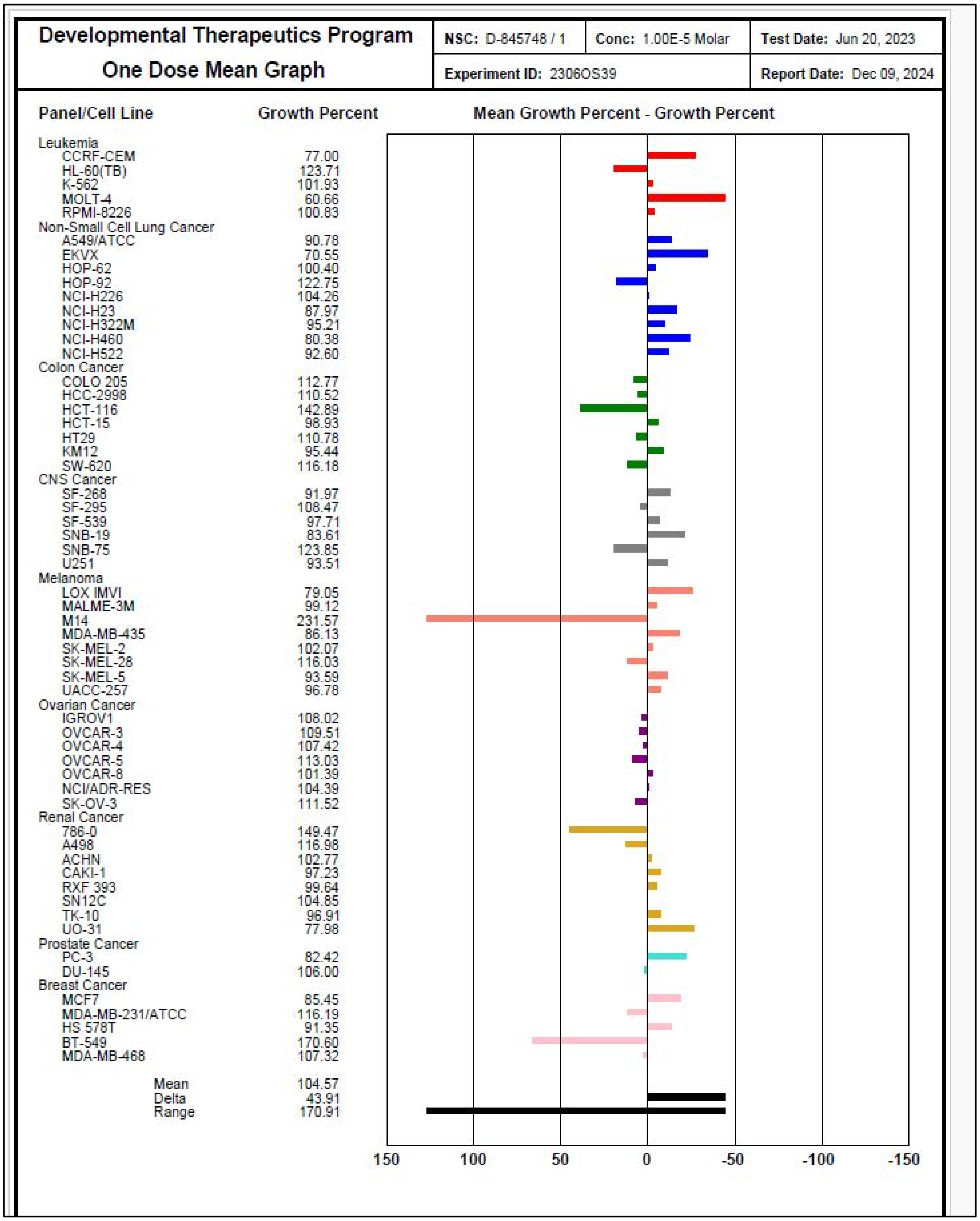

Figure S11. One dose anticancer screening of compound 6 (NCI, USA)

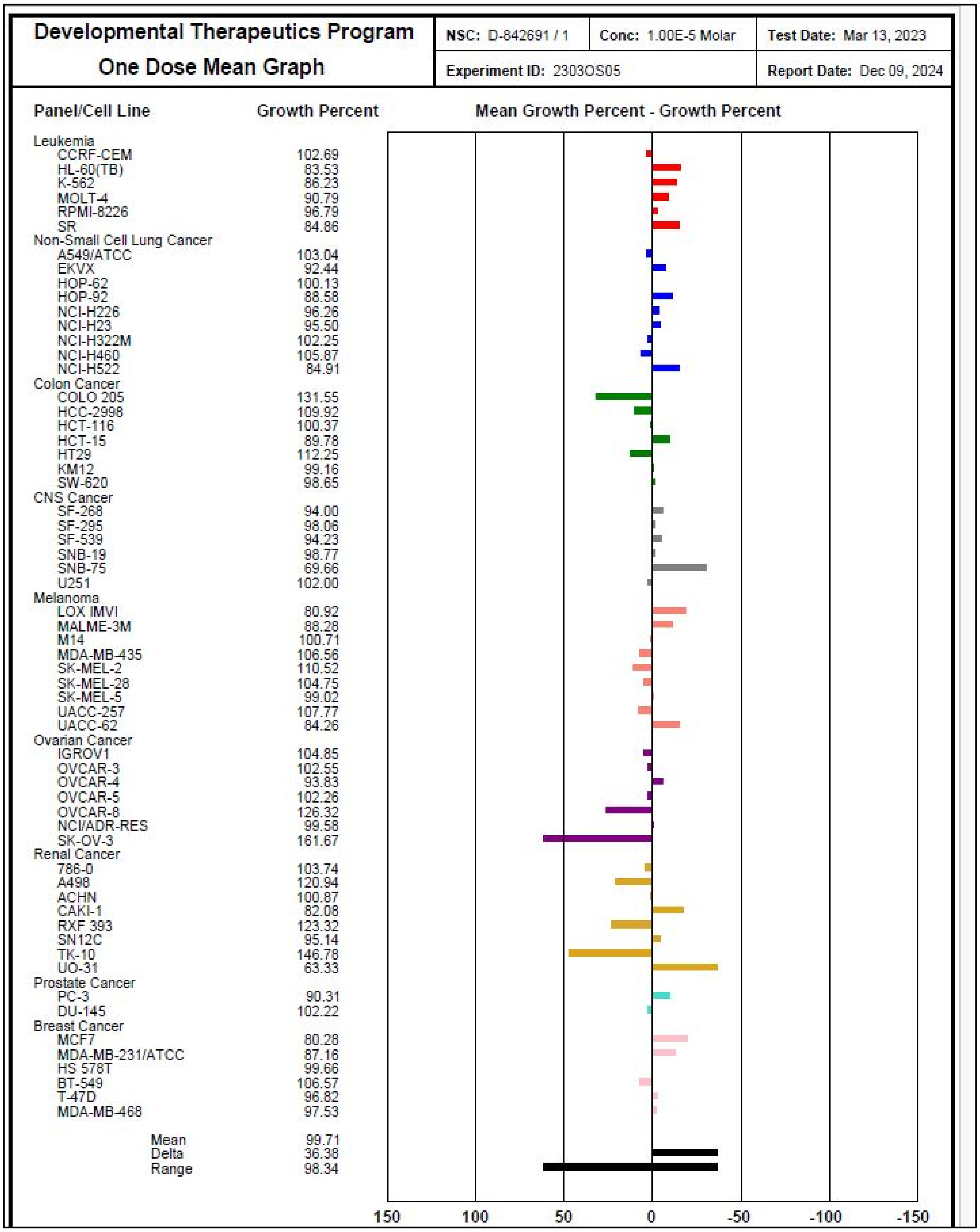

Figure S12. One dose anticancer screening of compound 7 (NCI, USA)

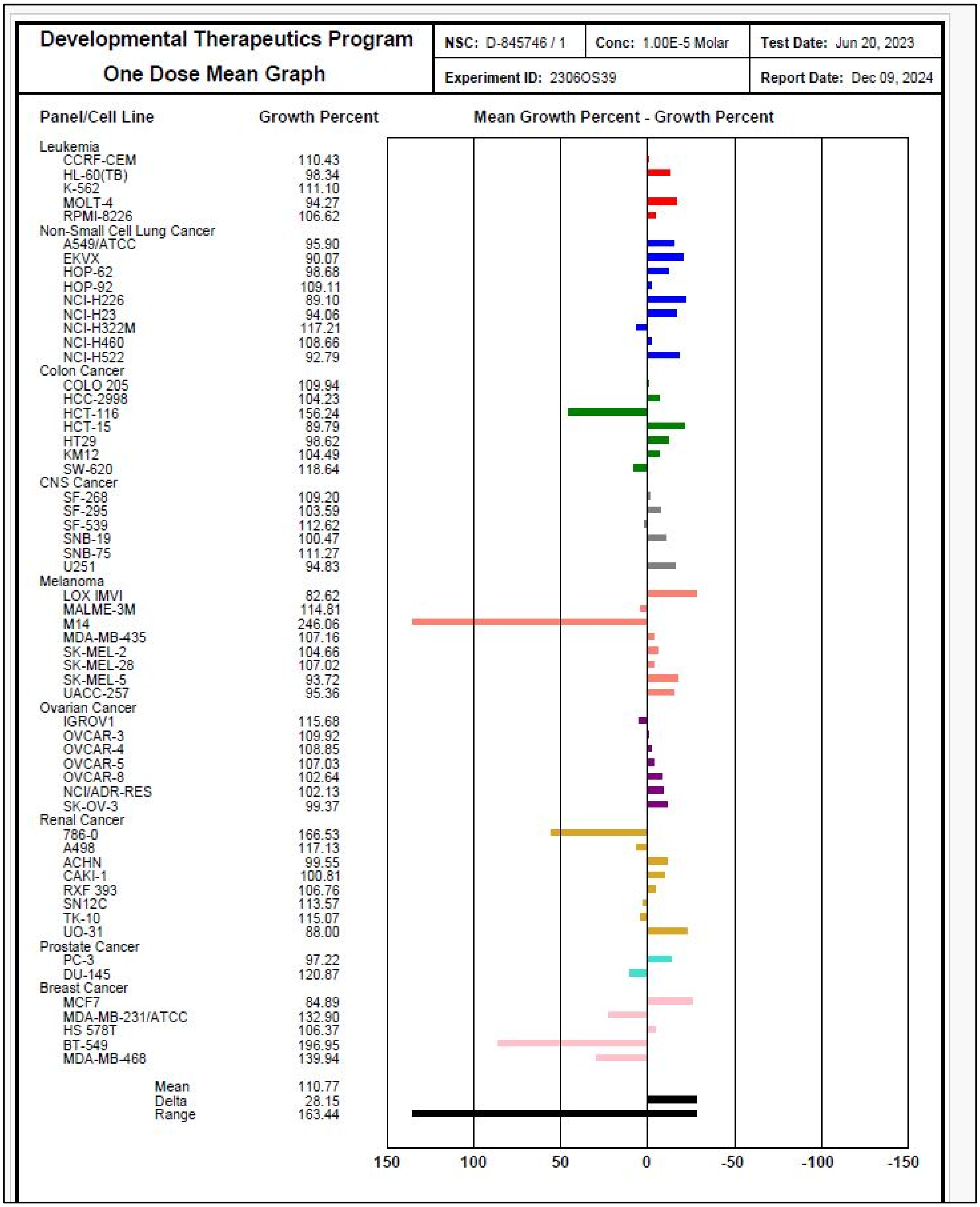

Figure S13. One dose anticancer screening of compound 8 (NCI, USA)

2.2. Cell viability assay of the target compound against melanoma LOX IMVI, colon HT29, and renal RXF393 cancer cell lines in addition to normal cell line WI 38.

(Detailed results)

Table S1. Cytotoxicity results of the target compound against melanoma LOX IMVI, colon HT29

Ser	Sample			cytotoxicity IC50 uM		SD
	code	M.W g/mol	F	HT-29	LOXIMVI	
1	SA-ST	458	---	24.3±1.29	9.55±0.51	
2	Doxorubicin	543.52	---	13.5±0.71	6.08±0.32	

Note that SA-ST refers to the compound 2 or the target compound

Table S2. Cytotoxicity results of the target compound against renal RXF393 cancer cell line

Ser	Sample			Cytotoxicity IC50 ug/ml	SD
	code	MW	cells	RXF 393	
1	SA-ST	---	---	7.01±0.39	
2	doxorubicin	---	---	13.54±0.82	

Note that SA-ST refers to the compound 2 or the target compound

Table S3. Cytotoxicity results of the target compound against WI-38 normal cell line

Ser	Sample			Cytotoxicity IC50 ug/ml	SD
	code	MW	cells	WI 38	
1	SA-ST	---	---	46.2±2.59	
2	doxorubicin			18.12±0.59	

Note that SA-ST refers to the compound 2 or the target compound

Table S4. Cell viability assay of the target compound against melanoma LOX IMVI, colon HT29, and renal RXF393 cancer cell lines in addition to normal cell line WI 38. **(Detailed results)**

researcher	assay	Date	cells
Dr.Hossam Aziz	MTT		

	Blank	CC	Sample No.		SA-ST//HT-29			sample no.		DOX/HT-29		
	1	2	3	4	5	6	7	8	9	10	11	12
A	B	C	100uM	25uM	6.3uM	1.6uM	0.4uM	100uM	25uM	6.3uM	1.6uM	0.4uM
B	B	C	100uM	25uM	6.3uM	1.6uM	0.4uM	100uM	25uM	6.3uM	1.6uM	0.4uM
C	B	C	100uM	25uM	6.3uM	1.6uM	0.4uM	100uM	25uM	6.3uM	1.6uM	0.4uM

ROBONIK P2000 Eia reader

Wave length: 450 nm

Reference: 630 nm

	1	2	3	4	5	6	7	8	9	10	11	12
A	0.001	0.549	0.218	0.275	0.331	0.392	0.445	0.177	0.242	0.303	0.365	0.441
B	0.001	0.552	0.225	0.262	0.325	0.388	0.461	0.185	0.258	0.323	0.383	0.431
C	0.001	0.546	0.212	0.274	0.339	0.379	0.4452	0.164	0.251	0.319	0.379	0.435
mean	4E-04	0.549	0.21833	0.2703	0.3317	0.3863	0.4504	0.1753	0.25033	0.315	0.376	0.436
%		`	39.7693	49.241	60.413	70.37	82.04	31.937	45.5981	57.377	68.43	79.36

SA-ST//HT-29

log conc.	% viability
2	39.77
1.4	49.24
0.8	60.41
0.19	70.37
-0.41	82.04
IC50=	

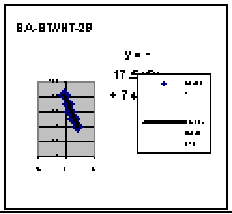

DOX/HT-29

log conc.	% viability
2	31.9369
1.3979	45.5981
0.7959	57.377
0.1931	68.4274
-0.4089	79.3564

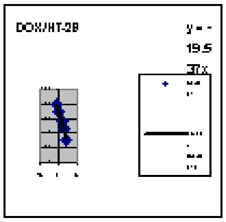

[redacted] [redacted] [redacted] [redacted] [redacted]

	Blank	CC	Sample No.		SA-ST/LOXIMVI			Sample No.		DOX/LOXIMVI		
	1	2	3	4	5	6	7	8	9	10	11	12
A	B	C	100uM	25uM	6.3uM	1.6uM	0.4uM	100uM	25uM	6.3uM	1.6uM	0.4uM
B	B	C	100uM	25uM	6.3uM	1.6uM	0.4uM	100uM	25uM	6.3uM	1.6uM	0.4uM
C	B	C	100uM	25uM	6.3uM	1.6uM	0.4uM	100uM	25uM	6.3uM	1.6uM	0.4uM

ROBONIK P2000 Eia reader

Wave length: 450 nm

Reference: 630 nm

	1	2	3	4	5	6	7	8	9	10	11	12
A	0.001	0.549	0.192	0.244	0.286	0.336	0.389	0.155	0.225	0.262	0.303	0.376
B	0.001	0.533	0.1193	0.249	0.284	0.335	0.385	0.159	0.219	0.274	0.318	0.381
C	0.001	0.527	0.184	0.252	0.293	0.333	0.384	0.161	0.224	0.259	0.322	0.369
mean	0.001	0.536	0.1651	0.2483	0.2877	0.3347	0.386	0.1583	0.22267	0.265	0.314	0.375
% Viability			30.7831	46.302	53.636	62.399	71.97	29.521	41.5165	49.41	58.61	69.98

SA-ST/LOXIMVI

log conc.	% viability

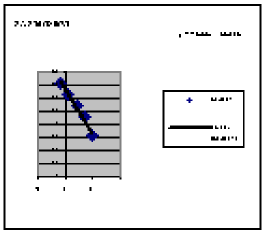

DOX/LOXIMVI

log conc.	% viability

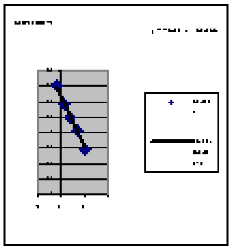

2	30.78
1.4	46.3
0.8	53.64
0.19	62.4
-0.41	71.97

2	29.5214
1.3979	41.5165
0.7959	49.4096
0.1931	58.6078
- 0.4089	69.9814

IC50= [redacted] [redacted] [redacted] [redacted]

	Blank	CC	Sample No.	SA-ST/WI38			
	1	2	3	4	5	6	7
A	B	C	100ug	25ug	6.25ug	1.6ug	0.4ug
B	B	C	100ug	25ug	6.25ug	1.6ug	0.4ug
C	B	C	100ug	25ug	6.25ug	1.6ug	0.4ug

ROBONIK P2000 eia reader

Wave length: 450 nm

Reference: 630 nm

	1	2	3	4	5	6	7
A	0.001	0.464	0.192	0.257	0.318	0.362	0.424
B	0.001	0.457	0.192	0.263	0.326	0.354	0.435
C	0.001	0.444	0.176	0.266	0.323	0.357	0.438
mean	0.001	0.455	0.1867	0.262	0.3223	0.3577	0.4323
% viability			41.026	57.5824	70.842	78.608	95.018

SA-ST/WI38

log conc.	% viability
2	41.0256
1.3979	57.5824
0.7959	70.8425
0.1931	78.6081
-0.409	95.0183

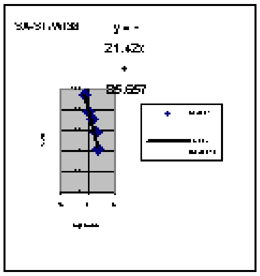

IC50= [redacted] [redacted] [redacted]

researcher	assay	Date	cells
Dr.Hossam Aziz	MTT		rxf393

	Blank	CC	Sample No.	SA-ST/rxf393				Sample No.	DOX/rxf393			
	1	2	3	4	5	6	7	8	9	10	11	12
A	B	C	100ug	25ug	6.25ug	1.6ug	0.4ug	100ug	25ug	6.25ug	1.6ug	0.4ug
B	B	C	100ug	25ug	6.25ug	1.6ug	0.4ug	100ug	25ug	6.25ug	1.6ug	0.4ug
C	B	C	100ug	25ug	6.25ug	1.6ug	0.4ug	100ug	25ug	6.25ug	1.6ug	0.4ug

ROBONIK P2000 eia reader

Wave length: 450 nm

Reference: 630 nm

	1	2	3	4	5	6	7	8	9	10	11	12
A	0.001	0.559	0.176	0.233	0.288	0.324	0.411	0.193	0.262	0.331	0.405	0.449
B	0.001	0.576	0.192	0.257	0.307	0.351	0.406	0.211	0.255	0.318	0.422	0.461
C	0.001	0.606	0.165	0.261	0.315	0.339	0.395	0.205	0.247	0.299	0.386	0.457
mean	0.00038	0.5803	0.1777	0.25033	0.3033	0.338	0.404	0.203	0.2547	0.316	0.4043	0.456
%		`	30.615	43.1361	52.269	58.242	69.615	34.98	43.883	54.451	69.673	78.52

SA-ST/rxf393

log conc.	% viability
2	30.6146
1.3979	43.1361
0.7959	52.2688
0.1931	58.2424
-0.409	69.6152

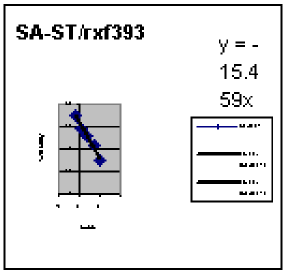

IC50= [redacted]

DOX/rxf393

log conc.	% viability
2	34.98
1.3979	43.883
0.7959	54.451
0.1931	69.673
-0.409	78.518

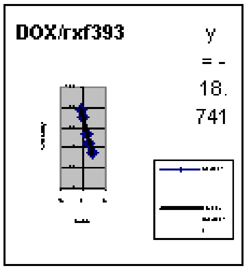

IC50= [redacted]

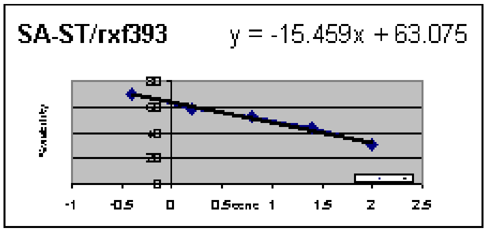

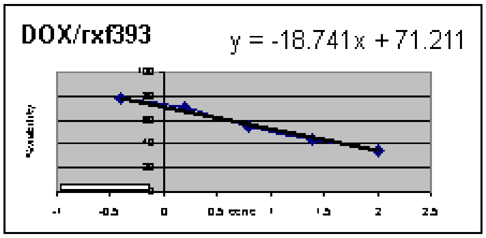

	Blank	CC	Sample No.		DOX/WI38			Sample No.				
	1	2	3	4	5	6	7	8	9	10	11	12
A	B	C	100uM	25uM	6.3uM	1.6uM	0.4uM					
B	B	C	100uM	25uM	6.3uM	1.6uM	0.4uM					
C	B	C	100uM	25uM	6.3uM	1.6uM	0.4uM					

ROBONIK P2000 Eia reader

Wave length: 450 nm

Reference: 630 nm

	1	2	3	4	5	6	7	8	9	10	11	12
A	0.001	0.535	0.211	0.249	0.288	0.344	0.394					
B	0.003	0.551	0.206	0.261	0.285	0.343	0.413					
C	0.001	0.549	0.211	0.252	0.283	0.346	0.411					
mean	0.002	0.545	0.20933	0.254	0.2853	0.3443	0.406	0	0	0	0	0

% Viability			38.4098	46.606	52.355	63.18	74.495	0	0	0	0	0

DOX/WI38

2	38.41
1.4	46.61
0.8	52.35
0.19	63.18
-0.41	74.5

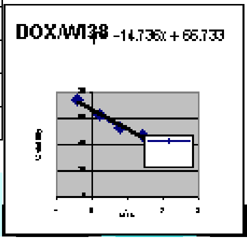

IC50= [redacted]

2.3. Evaluation of Carbonic anhydrase I, II, IV, and VII inhibition

Table S5. Evaluation of Carbonic anhydrase I of the target compound in comparison to acetazolamide

hCA-I

code	IC50	conc	log	%inh	T2	T1	ΔT	RFU2	RFU1	ΔRFU	slope	K.Activity
SA-ST		100	2	77.7	30	0	30	0.462	0	0.462	0.069	26.78261
		10	1	50.5	30	0	30	1.024	0	1.024	0.069	59.36232
		1	0	24.1	30	0	30	1.572	0	1.572	0.069	91.13043
		0.1	-1	14.1	30	0	30	1.779	0	1.779	0.069	103.1304
		0.01	-2	4.2	30	0	30	1.983	0	1.983	0.069	114.9565
EC				0	30	0	30	2.077	0	2.077	0.069	120

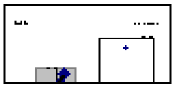

[redacted]

code	IC50	conc.uM	log conc	%inh	T2	T1	ΔT	RFU2	RFU1	ΔRFU	slope	K.Activity
AAZ		100	2	90.8	30	0	30	0.191	0	0.191	0.069	11.07246
		10	1	83.7	30	0	30	0.337	0	0.337	0.069	19.53623
		1	0	55.5	30	0	30	0.921	0	0.921	0.069	53.3913
		0.1	-1	39.6	30	0	30	1.251	0	1.251	0.069	72.52174
		0.01	-2	20.6	30	0	30	1.644	0	1.644	0.069	95.30435
EC				0	30	0	30	2.077	0	2.077	0.069	120

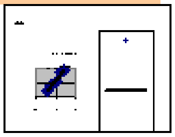

[redacted]

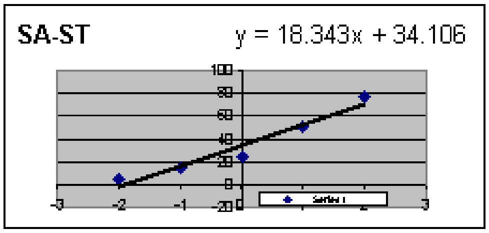

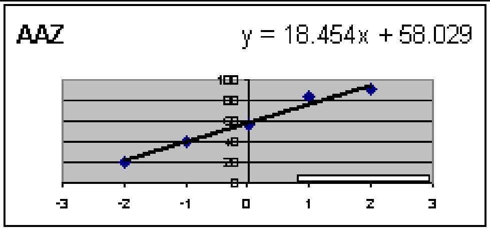

<u>Note that SA-ST refers to the target compound while AAZ refers to acetazolamide</u>

<u>Table S6.</u> Evaluation of Carbonic anhydrase II of the target compound in comparison to acetazolamide

hCA-II

code	IC50	conc.uM	log conc	%inh	T2	T1	ΔT	RFU2	RFU1	ΔRFU	slope	K.Activity
SA-ST		100	2	80.9	30	0	30	0.373	0	0.373	0.065	22.95385
		10	1	40.8	30	0	30	1.155	0	1.155	0.065	71.07692
		1	0	15.6	30	0	30	1.646	0	1.646	0.065	101.2923
		0.1	-1	3.54	30	0	30	1.881	0	1.881	0.065	115.7538
		0.01	-2	1.18	30	0	30	1.927	0	1.927	0.065	118.5846
EC				0	30	0	30	1.953	0	1.953	0.065	120

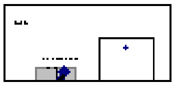

code	IC50	conc.uM	log conc	%inh	T2	T1	ΔT	RFU2	RFU1	ΔRFU	slope	K.Activity
AAZ		100	2	95.8	30	0	30	0.082	0	0.082	0.065	5.046154
		10	1	91.1	30	0	30	0.175	0	0.175	0.065	10.76923
		1	0	67	30	0	30	0.644	0	0.644	0.065	39.63077
		0.1	-1	40.9	30	0	30	1.152	0	1.152	0.065	70.89231
		0.01	-2	29.6	30	0	30	1.372	0	1.372	0.065	84.43077
EC				0	30	0	30	1.953	0	1.953	0.065	120

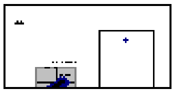

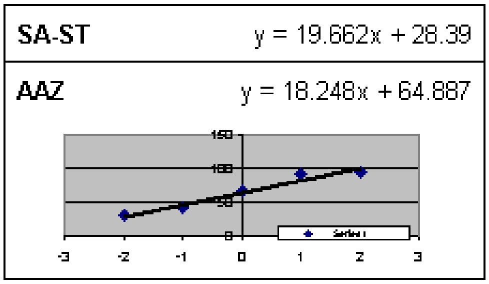

<u>Note that SA-ST refers to the target compound while AAZ refers to acetazolamide</u>

<u>Table S7.</u> Evaluation of Carbonic anhydrase IX of the target compound in comparison to acetazolamide

hCA-IX

code	IC50	conc.uM	log conc	%inh	T2	T1	ΔT	RFU2	RFU1	ΔRFU	slope	K.Activity
SA-ST		100	2	88	30	0	30	0.274	0	0.274	0.076	14.42105
		10	1	72.3	30	0	30	0.631	0	0.631	0.076	33.21053
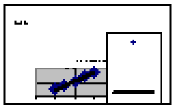		1	0	53.2	30	0	30	1.066	0	1.066	0.076	56.10526
		0.1	-1	36.9	30	0	30	1.438	0	1.438	0.076	75.68421
		0.01	-2	25.4	30	0	30	1.702	0	1.702	0.076	89.57895
EC				0	30	0	30	2.281	0	2.281	0.076	120

code	IC50	conc.uM	log conc	%inh	T2	T1	ΔT	RFU2	RFU1	ΔRFU	slope	K.Activity
AAZ		100	2	96.5	30	0	30	0.079	0	0.079	0.076	4.157895
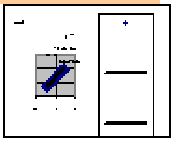		10	1	91.8	30	0	30	0.188	0	0.188	0.076	9.894737
		1	0	67	30	0	30	0.752	0	0.752	0.076	39.57895
		0.1	-1	47.7	30	0	30	1.193	0	1.193	0.076	62.78947
		0.01	-2	31.8	30	0	30	1.554	0	1.554	0.076	81.78947
EC				0	30	0	30	2.281	0	2.281	0.076	120

**SA-ST**	$y = 16.066x + 55.167$
AAZ	$y = 17.346x + 66.965$

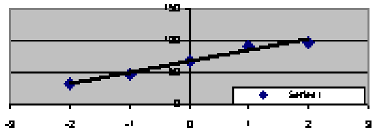

<u>Note that SA-ST refers to the target compound while AAZ refers to acetazolamide</u>

<u>Table S8.</u> Evaluation of Carbonic anhydrase XII of the target compound in comparison to acetazolamide

hCA-XII

code	IC50	conc.uM	log conc	%inh	T2	T1	ΔT	RFU2	RFU1	ΔRFU	slope	K.Activity
SA-ST		100	2	87.2	30	0	30	0.276	0	0.276	0.072	15.33333
		10	1	59.1	30	0	30	0.883	0	0.883	0.072	49.05556
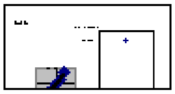		1	0	44.1	30	0	30	1.207	0	1.207	0.072	67.05556
		0.1	-1	21.7	30	0	30	1.691	0	1.691	0.072	93.94444
		0.01	-2	10.5	30	0	30	1.933	0	1.933	0.072	107.3889
EC				0	30	0	30	2.151	0	2.151	0.072	120

code	IC50	conc.uM	log conc	%inh	T2	T1	ΔT	RFU2	RFU1	ΔRFU	slope	K.Activity
AAZ		100	2	96.9	30	0	30	0.066	0	0.066	0.072	3.666667
		10	1	93.2	30	0	30	0.147	0	0.147	0.072	8.166667
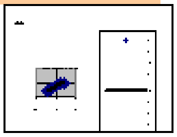		1	0	82.2	30	0	30	0.385	0	0.385	0.072	21.38889
		0.1	-1	58.1	30	0	30	0.906	0	0.906	0.072	50.33333
		0.01	-2	37.8	30	0	30	1.344	0	1.344	0.072	74.66667
EC				0	30	0	30	2.151	0	2.151	0.072	120

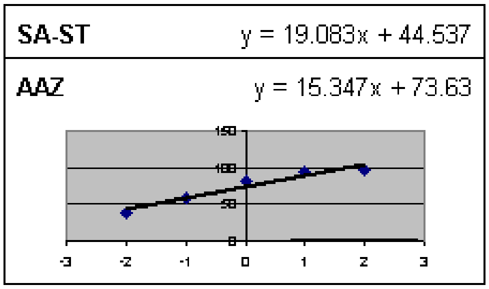

<u>Note that SA-ST refers to the target compound while AAZ refers to acetazolamide</u>
